# Early vs Deferred Non–Messenger RNA COVID-19 Vaccination Among Chinese Patients With a History of Inactive Uveitis: A Randomized Clinical Trial

**DOI:** 10.1001/jamanetworkopen.2022.55804

**Published:** 2023-02-14

**Authors:** Zhenyu Zhong, Qiuying Wu, Yuxian Lai, Lingyu Dai, Yu Gao, Weiting Liao, Guannan Su, Yao Wang, Chunjiang Zhou, Peizeng Yang

**Affiliations:** 1 The First Affiliated Hospital of Chongqing Medical University, Chongqing, China; 2 Chongqing Key Laboratory of Ophthalmology, Chongqing, China; 3 Chongqing Eye Institute, Chongqing, China; 4 Chongqing Branch of National Clinical Research Center for Ocular Diseases, Chongqing, China

## Abstract

**Importance:**

Improper host response to COVID-19 vaccines could trigger immune-mediated adverse events. The question remains whether COVID-19 vaccination should be postponed until complete remission in patients with uveitis, a preexisting immune-related condition.

**Objective:**

To compare recommendations for early and deferred COVID-19 vaccination with respect to uveitis outcomes.

**Design, Setting, and Participants:**

This open-label, randomized clinical trial at a large, specialized teaching center for uveitis care in China enrolled unvaccinated patients with inactive uveitis between August 10, 2021, and February 22, 2022, with follow-up to June 6, 2022.

**Interventions:**

Participants were randomly assigned to receive recommendation for early or deferred COVID-19 vaccination after complete remission of uveitis. Non–messenger RNA (non-mRNA) COVID-19 vaccines were available in China during the trial.

**Main Outcomes and Measures:**

The primary outcome was the time to symptomatic uveitis worsening during 3 months of follow-up. Secondary outcomes included uveitis activity and best-corrected visual acuity at 3 months.

**Results:**

Of the 543 participants (304 women [56.0%]; median age, 35 [IQR, 26-49] years), 262 were recommended for early vaccination and 281 for deferred vaccination. By month 3, 109 patients (41.6%) in the early group had been vaccinated compared with 14 (5.0%) in the deferred recommendation group. In the intention-to-treat population, the time to symptomatic uveitis worsening was shorter in the early group than in the deferred group (hazard ratio, 1.68 [95% CI, 1.09-2.59]; *P* = .01 by log-rank test). Changes in anterior chamber cells, vitreous haze, and best-corrected visual acuity from baseline to month 3 appeared similar in the 2 groups in the evaluable population after the month 3 in-person visit.

**Conclusions and Relevance:**

In this randomized clinical trial of patients with inactive uveitis, recommendation for early non-mRNA COVID-19 vaccination resulted in a higher incidence of self-reported symptomatic uveitis worsening with possible reporting bias compared with recommendation for deferred vaccination, but no adverse effects were observed in disease and visual prognosis at 3 months. These findings would be useful to guide the individual timing choices of non-mRNA COVID-19 vaccination in this clinically vulnerable population.

**Trial Registration:**

Chinese Clinical Trial Registry: ChiCTR2100049467

## Key Points

**Question:**

Is there any difference in uveitis outcomes between patients with inactive disease given recommendations for early and deferred non–messenger RNA (mRNA) COVID-19 vaccination?

**Findings:**

In this open-label, randomized clinical trial involving 543 patients, recommendation for early vaccination resulted in an increased incidence of self-reported worsening of symptomatic uveitis compared with deferred vaccination, but no differences were observed in disease and visual prognosis at 3 months.

**Meaning:**

These findings suggest that recommendation for early non-mRNA COVID-19 vaccination in patients with inactive uveitis may lead to subjective uveitis symptoms but no adverse effects on disease and visual prognosis at 3 months.

## Teaser

This randomized clinical trial compares recommendations for early and deferred non–messenger RNA COVID-19 vaccination with respect to ocular outcomes among patients with inactive uveitis.

## Introduction

COVID-19 vaccines remain the cornerstone of effective prevention of severe illness and death due to SARS-CoV-2.^1,2,3^ Billions of doses of COVID-19 vaccines have been administered worldwide, while vaccine uptake in certain medically vulnerable populations is frequently lower than expected.^4,5^ Some studies reported that improper host response to COVID-19 vaccines could trigger rare and severe immune-mediated adverse events, such as thrombotic thrombocytopenia and Guillain-Barré syndrome.^6,7^ Reasons for undervaccination, especially among those with preexisting autoimmune conditions, may be related to fear of similar adverse effects and lack of appropriate recommendations on the need for vaccination.^8^

Uveitis is an immune-related disorder characterized by vision-threatening intraocular inflammation, which has a wide association with systemic inflammatory conditions.^9,10,11^ The symptoms and signs of uveitis often sensitively reflect a certain level of activity of systemic inflammatory diseases and affect the quality of life of patients.^9,10,12,13,14^ Systemic corticosteroids and noncorticosteroid immunomodulatory agents are frequently used to treat uveitis, but these therapies would impose a higher risk of SARS-CoV-2 infection and severe illness.^15,16^ COVID-19 vaccination was found to provide some extent of protection against SARS-CoV-2 infection among individuals who were taking glucocorticoids and immunosuppressive agents.^17^ Nevertheless, observational studies indicated that COVID-19 vaccination was associated with an increased incidence of new-onset or relapse of uveitis.^18,19,20,21^ Due to concerns about the potential worsening activity of uveitis, patient counseling on the appropriate timing of COVID-19 vaccination frequently occurs during patient-clinician encounters, particularly when a patient has achieved and maintained inactive disease with medication use. Guidance from clinicians would be expected to have an impact on public health, such as affecting the coverage of COVID-19 immunization, the course of population immunity, and the protection of special groups. However, evidence supporting which timing treating clinicians should recommend is lacking. In this randomized clinical trial involving patients with inactive uveitis, we compare recommendations for early and deferred non–messenger RNA (mRNA) COVID-19 vaccination with respect to uveitis outcomes.

## Methods

### Trial Design

This open-label randomized clinical trial was conducted at The First Affiliated Hospital of Chongqing Medical University, Chongqing, China. The hospital has set up a large, specialized teaching center for uveitis care and has treated 15 373 patients with uveitis across mainland China as of 2018.^10^ The trial protocol (found in Supplement 1) was approved by the ethics committee of The First Affiliated Hospital of Chongqing Medical University. The participants or the parents or legal guardians of minors provided written informed consent, and children gave assent when appropriate. Participants were recruited between August 10, 2021, and February 22, 2022, and were followed up until June 6, 2022. The First Affiliated Hospital of Chongqing Medical University oversaw the trial conduct. All trial procedures adhered to the tenets of the Declaration of Helsinki.^22^ This study followed the Consolidated Standards of Reporting Trials (CONSORT) reporting guideline.

### Trial Population

Eligible participants were 12 years or older, had not yet received COVID-19 vaccines, and were diagnosed with any form of uveitis in an inactive status.^23,24,25^ Uveitis inactivity was defined as an anterior chamber cell grade of 0.5+ or less, a vitreous haze grade of 0.5+ or less, and no active inflammatory choroidal or retinal vascular lesions in both eyes. Anterior chamber cells were graded according to the Standardization of Uveitis Nomenclature criteria on a range from 0 to 4, with higher values indicating greater severity of anterior segment inflammation of the eye.^25^ Vitreous haze was graded based on the National Eye Institute criteria adapted by the Standardization of Uveitis Nomenclature working group on a scale from 0 to 4, with higher values indicating worse activity of posterior segment inflammation.^25,26^ We excluded individuals in complete remission of uveitis, defined as an inactive condition lasting for 3 months or more after discontinuing all treatments.^25^ We also excluded those with any known vaccine contraindications, including a history of anaphylaxis to any component of the vaccine, a body temperature higher than 38.5°C, and pregnancy. All participants were Chinese, and ethnicity data were routinely collected from their resident identity cards (including Han, Dong, Hani, Hui, Li, Lisu, Man, Mongolian, Miao, Tibetan, Tujia, Uygur, Yi, and Zhuang peoples).

### Randomization, Assignment, and Follow-up

Participants underwent simple randomization to receive the recommendation for early or deferred COVID-19 vaccination in a 1:1 ratio. Random assignment sequence was computer-generated, and individual allocations were known by telephone from a designated staff who kept the sequence and had no involvement in other parts of the trial. Patients and treating clinicians were aware of the trial assignment.

Patients in the early vaccination group were recommended to receive COVID-19 vaccines as soon as possible, while patients in the deferred vaccination recommendation group were recommended to receive COVID-19 vaccines after the complete remission of uveitis. Our clinic did not provide COVID-19 vaccination service. We assessed patients’ uveitis status and only gave the recommendation to patients. Non-mRNA COVID-19 vaccines were free of charge and available in the community. During the trial, 2 inactivated SARS-CoV-2 vaccines, CoronaVac (Sinovac Biotech) and BBIBP-CorV (Sinopharm), and 1 protein subunit vaccine, ZF2001 (Anhui Zhifei Longcom Biopharmaceutical Co, Ltd), were available under emergency use authorization among people 12 years or older in China.^27,28,29^ Patients’ COVID-19 vaccination status was verified via access to the Health Code System managed by the local health administration, where vaccine information about date, site, and type of each dose administered were recorded in real time.

Routine follow-up encounters occurred every month by telephone, and an in-person clinic visit was scheduled at month 3. At the telephone follow-up encounters, patients were asked about adherence to the recommendation, reasons for nonadherence, experiences of systemic adverse events, and uveitis symptoms, including the presence and duration of new-onset eye redness, eye pain, decreased vision, light sensitivity, and floaters. These queries were administered in a standardized telephone interview from the trial call center. Information on missed encounters could be completed in subsequent encounters provided that participants were brought back. In addition, all participants were repeatedly encouraged to follow the assigned vaccination recommendation during the telephone call. If the aforementioned symptoms or systemic adverse events were reported, patients would be instructed to return to the study site early or visit local hospitals for further clinical evaluation. We asked patients to provide their medical records from their local hospitals if any. Patients would receive best medical judgment and proper treatment, and intervention with trial recommendations would be terminated. At the month 3 in-person visit, patients underwent additional clinical evaluations, including slitlamp biomicroscopy, ophthalmoscopy, measurement of best-corrected visual acuity (BCVA), and other auxiliary examinations according to clinical needs.

### Outcomes

The primary outcome was the time to worsening of symptomatic uveitis, defined by 1 of the following new-onset symptoms occurring in at least 1 eye and lasting for at least 2 days: eye redness, eye pain, decreased vision, light sensitivity, or floaters, as confirmed by blinded review by an adjudication committee. The adjudication committee consisted of 3 independent clinicians, and the outcome was achieved with a majority of 2. Key secondary outcomes included proportion of COVID-19 vaccination, proportion of adherence to recommendation, proportion of each worsening symptom, proportion of 2-grade increase in anterior chamber cells from baseline to month 3, proportion of 2-grade increase in vitreous haze from baseline to month 3, change in BCVA from baseline to month 3, and incidence of systemic adverse events. The original protocol also included secondary outcomes concerning clinically confirmed uveitis worsening, hospitalization, and treatment, which were determined based on in-person clinical evaluation when patients returned to the study site or visited local hospitals with symptomatic uveitis worsening. During the execution of the trial, most cases with worsening symptoms did not seek medical services and assessment at our study center, nor did they provide medical records to show their local hospital visits, possibly due to their mild diseases, self-care, remote living, or COVID-19 travel restrictions. Therefore, these secondary outcomes were not reported in terms of data unavailability.

### Statistical Analysis

Assuming that the cumulative incidence would be 10% in the deferred vaccination group during the month 3 follow-up and the proportion dropping out in both groups would be 15%, we estimated that an overall sample of 1314 participants would provide 80% power to detect a hazard ratio (HR) for early vaccination recommendation of 1.50 for the primary outcome compared with deferred vaccination recommendation, at a 2-sided significance level of *P* = .05. The target of enrollment was not met because of the absence of eligible unvaccinated patients in the later period of trial. A decision was made to suspend enrollment on February 28, 2022, as the full vaccination rate improved to 86.6% in China.^30^

The primary analysis was performed according to the intention-to-treat principle in all randomized participants, regardless of their adherence to the assigned recommendation. Outcome times were calculated from randomization to the encounter reporting the end point event or the last follow-up. Time-to-event data were analyzed using the Kaplan-Meier method, with trial groups compared using a log-rank test and HRs calculated using Cox proportional hazards regression. The proportional hazards assumption was met as confirmed by inspection of log-log survival plot (eFigure in Supplement 2). With the introduction of the Health Code System to verify the vaccination status, we subsequently identified 32 patients who concealed or mistakenly reported their COVID-19 vaccination history at enrollment. These patients were excluded from a modified intention-to-treat analysis as they were vaccinated before randomization. Secondary analysis populations also included the per-protocol population, the population who completed telephone follow-up, and the population with evaluable data (hereinafter referred to as the evaluable population) at the month 3 follow-up (eTable 1 in Supplement 2). We performed as-treated analyses and instrumental variable analyses (eMethods in Supplement 2) to examine the actual effect of COVID-19 vaccination on the primary outcome.^31,32^ We performed 1 prespecified sensitivity analysis with multiple imputation for nonresponse and several post hoc analyses (eTable 2 in Supplement 2). Prespecified subgroup analyses were performed by testing interaction effects between each factor and trial group in the regression model.

Binary outcomes are presented as numbers with proportions. We calculated 95% CIs for between-group differences in proportions using the Wilson score method.^33^ Changes in BCVA were analyzed with the generalized estimating equation with identity link, adjusted for baseline value and the correlation between eyes of the same patient. To account for data missing in secondary outcomes, analyses were repeated in the month 3 in-person evaluable population by using a weighted model, where weights were based on the inverse of the modeled nonmissing probability.^34^ A 2-sided *P* < .05 was considered statistically significant for the primary analysis. In secondary and sensitivity analyses, 95% CIs were not adjusted for multiplicity and should not be used for drawing formal clinical inferences. Analyses were performed with SPSS Statistics, version 25.0 (IBM Corporation), and R, version 3.5.0 (R Project for Statistical Computing).

## Results

### Patients

**Figure 1.**
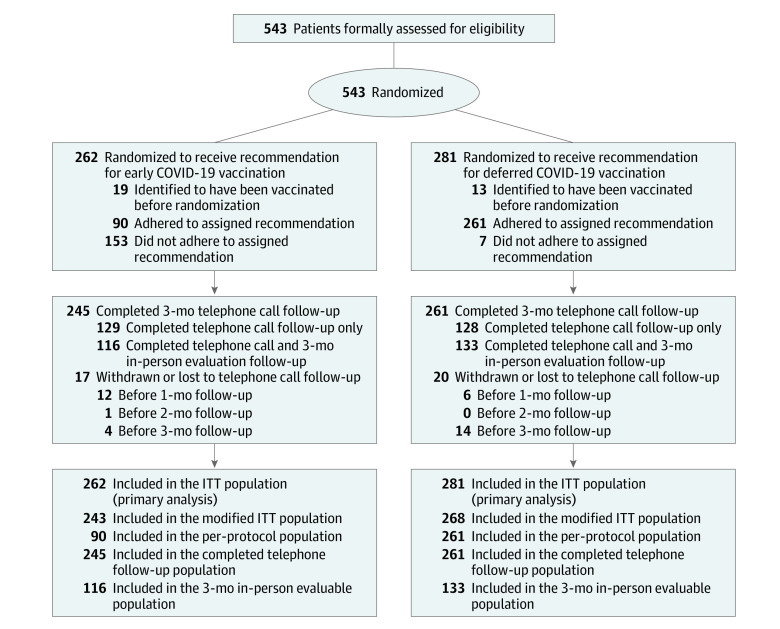
Randomization, Follow-up, and Analysis Populations The intention-to-treat (ITT) population included 543 patients who underwent randomization. The modified ITT population excluded 32 persons who were identified as being vaccinated before randomization. The per-protocol population included the patients in the modified ITT population except 160 persons who did not adhere to assigned recommendation. The completed telephone follow-up population included the patients in the ITT population, except 37 persons who did not complete 3-month telephone call follow-up. The month 3 in-person evaluable population included the patients in the ITT population except 294 persons who did not complete month 3 in-person follow-up.

A total of 543 patients were formally assessed for eligibility and enrolled. The median age was 35 (IQR, 26-49) years; 304 patients (56.0%) were female and 239 (44.0%) were male. Two hundred sixty-two patients were recommended for early COVID-19 vaccination and 281 for deferred vaccination. The baseline characteristics were similar between 2 groups (Table 1). Before June 6, 2022, 506 patients (93.2%) completed the 3-month follow-up by telephone, and 249 (45.9%) completed the in-person follow-up (Figure 1). No participants were infected with SARS-CoV-2 during the trial.

**Table 1.** Baseline Characteristics of the Intention-to-Treat Population

Characteristic	Vaccination recommendation group^a^	
	Early (n = 262)	Deferred (n = 281)
Age, median (IQR), y	36 (26-49)	35 (26-48)
Sex		
Female	149 (56.9)	155 (55.2)
Male	113 (43.1)	126 (44.8)
Ethnic group		
Chinese Han	248 (94.7)	265 (94.3)
Chinese minority group^b^	14 (5.3)	16 (5.7)
Duration of uveitis, median (IQR), mo^c^	31 (16-62)	35 (17-68)
Etiology of uveitis		
Noninfectious	244 (93.1)	265 (94.3)
Infectious	18 (6.9)	16 (5.7)
Type of uveitis		
Anterior	74 (28.2)	71 (25.3)
Intermediate, posterior or panuveitis	188 (71.8)	210 (74.7)
BCVA in the better-seeing eye^c^		
≥20/63	239 (91.2)	244 (86.8)
<20/63 but ≥20/400	18 (6.9)	33 (11.7)
<20/400	1 (0.4)	4 (1.4)
No. of flares in the past 12 mo^d^		
0	192 (73.3)	203 (72.2)
1	43 (16.4)	42 (14.9)
2	15 (5.7)	20 (7.1)
≥3	10 (3.8)	14 (5.0)
Duration of uveitis inactivity, mo^d^		
≤6	17 (6.5)	20 (7.1)
>6 but ≤12	51 (19.5)	56 (19.9)
>12	192 (73.3)	203 (72.2)
Medical history and comorbidities		
HBV infection	45 (17.2)	40 (14.2)
Hypertension	6 (2.3)	10 (3.6)
Tuberculosis	9 (3.4)	4 (1.4)
Diabetes	2 (0.8)	1 (0.4)
Cardiovascular and cerebrovascular diseases	2 (0.8)	1 (0.4)
Asthma	2 (0.8)	0
Cancer	1 (0.4)	1 (0.4)

Abbreviations: BCVA, best-corrected visual acuity; HBV, hepatitis B virus.

^a^ Unless otherwise specified, data are expressed as No. (%) of patients. Percentages have been rounded and may not total 100.

^b^ Includes Chinese Dong, Hani, Hui, Li, Lisu, Man, Mongolian, Miao, Tibetan, Tujia, Uygur, Yi, and Zhuang peoples.

^c^ Data were missing for 4 patients in the early vaccination group.

^d^ Data were missing for 2 patients in the early vaccination group and 2 patients in the deferred vaccination group.

### COVID-19 Vaccine Uptake

By month 3, 109 patients (41.6%) of the patients assigned to early vaccination recommendation had received at least 1 dose of non-mRNA COVID-19 vaccine compared with 14 (5.0%) of those assigned to a deferred vaccination recommendation (Table 2). The full vaccination (not including boosters) was completed by 90 patients (34.4%) in the early vaccination group and 12 (4.3%) in the deferred vaccination group. Of the total 123 patients who received at least 1 dose of vaccine by 3 months, 118 (95.9%) received inactivated SARS-CoV-2 vaccines (eTable 3 in Supplement 2). Irrespective of receiving the vaccine before or after randomization, patients whose vaccination status was in line with their assigned recommendation by month 3 accounted for 109 (41.6%) of those in the early vaccination group and 263 (93.6%) of those in the deferred vaccination group (Table 2). The proportions of unvaccinated patients who were advised to get the vaccine during the trial appeared similar in the 2 groups (153 of 262 [58.4%] vs 2 of 4 [50.0%]) (eTable 4 in Supplement 2). This was because all 262 patients in the early vaccination group were advised to get the vaccine but only 4 patients who achieved complete remission of uveitis were so advised in the deferred vaccination group.

**Table 2.** Secondary Outcomes

Outcome	Vaccination recommendation group^a^		Difference, % (95% CI)
	Early (n = 262)	Deferred (n = 281)	
Vaccination and adherence by month 3			
Received ≥1 dose	109 (41.6)	14 (5.0)	36.6 (30.0 to 43.0)
Completed vaccination schedule^b^	90 (34.4)	12 (4.3)	30.1 (23.8 to 36.3)
Vaccination status was in line with recommendation	109 (41.6)	263 (93.6)	−52.0 (−58.2 to −45.0)
Not vaccinated and advised to get the vaccine, No./total No. (%)^c^	153/262 (58.4)	2/4 (50.0)	8.4 (−27.1 to 43.9)
Symptomatic uveitis worsening according to reasons^d^			
Eye redness	10 (3.8)	9 (3.2)	0.6 (−2.7 to 4.0)
Eye pain	11 (4.2)	11 (3.9)	0.3 (−3.2 to 3.9)
Decreased vision	31 (11.8)	25 (8.9)	2.9 (−2.2 to 8.2)
Light sensitivity	4 (1.5)	1 (0.4)	1.2 (−0.7 to 3.5)
Floaters	12 (4.6)	1 (0.4)	4.2 (1.7 to 7.5)
Ocular condition at month 3, No./total No. (%)^e^			
2-Grade increase in anterior chamber cells	10/116 (8.6)	13/133 (9.8)	−1.2 (−8.5 to 6.5)
2-Grade increase in vitreous haze	1/116 (0.9)	3/133 (2.3)	−1.4 (−5.6 to 2.7)
Change in BCVA, mean (SE), LogMAR^f^	−0.006 (0.010)	0.006 (0.016)	−0.012 (−0.049 to 0.025)
Systemic event			
Any systemic event	4 (1.5)	11 (3.9)	−2.4 (−5.5 to 0.5)
Serious adverse event	0	3 (1.1)	−1.1 (−3.1 to 0.5)
Death	0	1 (0.4)	−0.4 (−2.0 to 1.1)
Pneumonia	0	2 (0.7)	−0.7 (−2.6 to 0.8)

Abbreviations: BCVA, best corrected visual acuity; LogMAR, log of the minimum angle of resolution.

^a^ Unless otherwise indicated, data are expressed as No. (%) of patients.

^b^ COVID-19 vaccination schedule included 2 primary doses of inactivated vaccines (CoronaVac [Sinovac Biotech] and BBIBP-CorV [Sinopharm]) or 3 doses of a protein subunit vaccine (ZF2001 [Anhui Zhifei Longcom Biopharmaceutical Co, Ltd]).

^c^ In the deferred vaccination group, 4 patients had complete remission of uveitis during the study period and should have been vaccinated according to the trial recommendation.

^d^ Each patient may have had 2 or more reasons.

^e^ These outcomes were assessed in the month 3 in-person evaluable population (n = 249) relative to the baseline condition.

^f^ Analyzed by eye with the generalized estimating equation to account for baseline values and the correlation between eyes of the same patient. Visual acuity data are expressed as LogMAR scores, with higher values indicating poorer visual acuity.

### Primary Outcome

**Figure 2.**
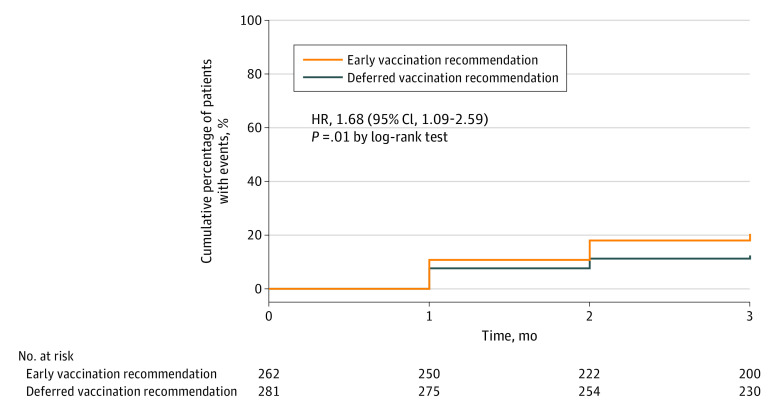
Kaplan-Meier Estimates of the Time to Symptomatic Uveitis Worsening HR indicates hazard ratio.

**Figure 3.**
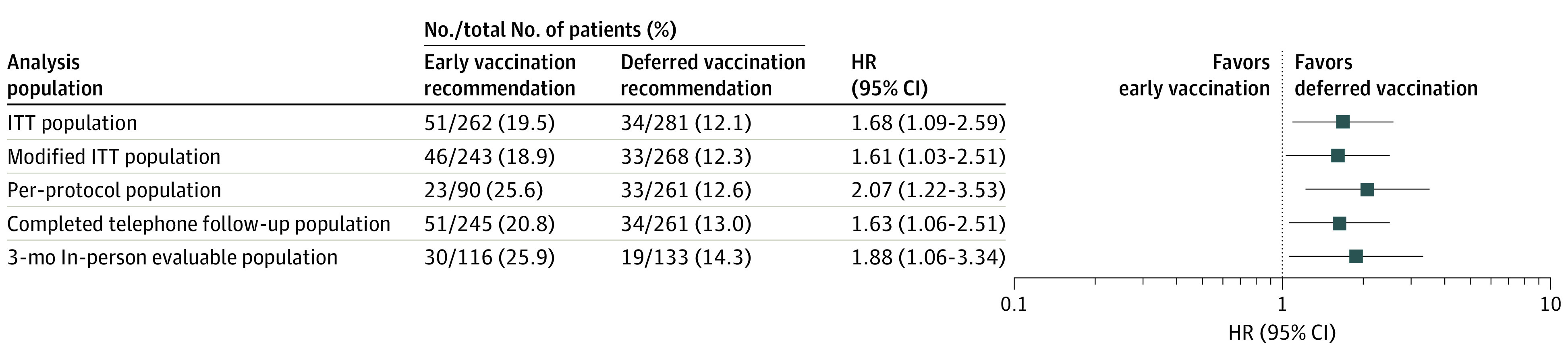
Estimates of Primary Outcome Across Analysis Populations HR indicates hazard ratio; ITT, intention-to-treat.

Symptomatic uveitis worsening occurred in 51 patients (19.5%) recommended for early COVID-19 vaccination and in 34 (12.1%) recommended for deferred COVID-19 vaccination (between-group difference, 7.4% [95% CI, 1.2%-13.6%]). In the intention-to-treat analysis, the time to symptomatic uveitis worsening was shorter in the early vaccination group than in the deferred vaccination group (HR, 1.68 [95% CI, 1.09-2.59]; *P* = .01 by the log-rank test) (Figure 2). The finding was consistent across analysis populations (Figure 3), prespecified subgroups (eTable 5 in Supplement 2), sensitivity analyses (eTable 6 in Supplement 2), and as-treated analyses (eTable 3 in Supplement 2). The higher incidence of symptomatic uveitis worsening in the early vaccination group could be attributed to more COVID-19 vaccinations (instrumental variable estimation of causal HR of vaccination, 4.12 [95% CI, 1.26-13.46]) (eTable 7 in Supplement 2). Among those 85 patients with symptomatic uveitis worsening, it occurred in 27 patients after receiving COVID-19 vaccines. Symptomatic uveitis worsening post COVID-19 vaccination occurred in 26 cases (9.9%) in the early vaccination group and 1 case (0.4%) in the deferred vaccination group. A similar incidence of symptomatic uveitis worsening occurring before COVID-19 vaccination was reported in the 2 groups (early vs deferred: 25 of 262 [9.5%] vs 33 of 281 [11.7%]). When limited to the unvaccinated participants (eTable 8 in Supplement 2), the primary outcome was similar in the 2 groups (HR, 1.30 [95% CI, 0.77-2.22]).

### Secondary Outcomes

Patients in the early vaccination group more frequently reported new onset of floaters as one of the reasons for symptomatic uveitis worsening (difference, 4.2% [95% CI, 1.7%-7.5%]). The ocular conditions, including changes in anterior chamber cells, vitreous haze, and BCVA from baseline to month 3, appeared similar between the 2 groups in the month 3 in-person evaluable population (Table 2), even after accounting for nonadherence (eTable 9 in Supplement 2) and nonresponse (eTable 10 in Supplement 2). One death and 2 cases of pneumonia occurred in unvaccinated participants (Table 2). No other serious safety signals were observed during the 3-month follow-up (eTables 11 and 12 in Supplement 2).

## Discussion

This open-label, randomized clinical trial showed that recommendation for early non-mRNA COVID-19 vaccination to patients with inactive uveitis led to earlier self-reported symptomatic uveitis worsening than the recommendation for deferred vaccination. We found no evidence that uveitis activity and visual acuity at 3 months differed substantially according to recommendation type.

This trial extends prior observations linking COVID-19 vaccination with an increased risk of newly active or worsening uveitis^18,19,20,21^ and in particular provides randomized evidence for such an effect. However, our findings suggest that COVID-19 vaccination might temporarily affect the symptoms but would not modify the disease and visual prognosis at 3 months. A reasonable explanation may be that uveitis worsening after COVID-19 vaccination appears to be acute and transient, usually responds to corticosteroid therapy, and may be quickly controlled.^18,35,36,37^

As a subjective measure, the primary outcome could at least partially reflect subjective feelings about the disease condition and certain levels of quality of life. However, participants were aware of their vaccination status, and those concerned about vaccination could have an exacerbated experience of their symptoms. Our trial detected the significant effects of recommending early COVID-19 vaccination on patient’s symptoms. However, the between-group mean difference (7.4% [95% CI, 1.2%-13.6%]) in incidence of symptomatic uveitis worsening was subtle, and it would be possible to have reporting bias from people who received COVID-19 vaccines as large as that would negate the currently reported significant difference in the primary outcome. More importantly, no adverse effects were observed in disease and visual prognosis at 3 months. The inconsistency between the primary and secondary outcomes also suggested the possibility of reporting bias incurred by psychological factors related to subjective assessment of the primary outcome. These findings highlight that, in clinical practice, more psychological reassurance should be provided to patients before or after COVID-19 vaccination, especially those worried about the adverse effects of COVID-19 vaccine. The timing choices for COVID-19 vaccination in inactive uveitis may be primarily based on the individual risk of SARS-CoV-2 infection and severe COVID-19 illness as well as patient’s expectations and preferences rather than on anticipated differences in the disease and visual prognosis of uveitis.

### Limitations

This study has some limitations. First, interpretation of the outcomes was limited by the open nature of the trial, especially when interpreting results for a subjective outcome in the context of COVID-19 vaccination. Moreover, all symptoms were based on self-report, and we lacked data regarding clinical evaluation to confirm disease worsening. The possibility that the greater reporting bias in patients receiving COVID-19 vaccines may lead to the slightly higher incidence rather than the vaccine increasing inflammation cannot be ruled out. Second, our sample size fell short of expectations due to the lack of eligible unvaccinated patients in China, although this sample size was powered to detect the difference in the primary outcome. Third, a number of patients were not included in the month 3 in-person evaluable population. Nevertheless, this population was only related to several secondary outcomes, in which data showed materially small between-group differences in the point estimate. Moreover, results were similar after accounting for data missing with inverse probability weighting. Fourth, we could not precisely define which uveitis worsening was caused by COVID-19 vaccination. Instead, we only examined the excess risk of symptomatic worsening in the early vaccination group compared with the deferred one, with randomization leading to the balanced background risk between the 2 groups. To mitigate the interference of other factors unrelated to vaccination, we chose a 3-month observation period for vaccine uptake and uveitis worsening because a later onset of uveitis flare was less likely to be related to COVID-19 vaccination.^18,19,38^ However, such a short-term follow-up compromised the representativeness of long-term disease and visual prognosis. In addition, due to the vaccine availability in China, inactivated vaccines made up 95.9% of those administered, and mRNA vaccines were not included in the trial, which might limit the generalizability of the findings.

## Conclusions

In this randomized clinical trial of patients with inactive uveitis, recommendation for early non-mRNA COVID-19 vaccination resulted in a higher incidence of self-reported symptomatic uveitis worsening with possible reporting bias compared with recommendation for deferred vaccination, but no adverse effects were observed in disease and visual prognosis at 3 months. These findings would be useful to guide the individual timing choices of non-mRNA COVID-19 vaccination in this clinically vulnerable population.

## Notes

Supplement 1.
Trial Protocol

Supplement 2. **eMethods.** Instrumental Variable Analyses **eTable 1.** Definition of Analysis Population **eTable 2.** Summary of Sensitivity Analyses **eTable 3.** As-Treated Analysis of Primary Outcome **eTable 4.** Reasons for Nonadherence to Vaccine Uptake as Recommended **eTable 5.** Subgroup Analysis of Primary Outcome **eTable 6.** Results of Sensitivity Analyses of Primary Outcome **eTable 7.** Instrumental Variable Analysis **eTable 8.** Comparative Analysis of Primary End Point Event Among Unvaccinated Participants **eTable 9.** Per Protocol Analysis of Ocular Condition at Month 3 in In-Person Evaluable Population **eTable 10.** Inverse Probability Weighting for Evaluating Ocular Condition at Month 3 to Account for Missing Data **eTable 11.** Definition of Systemic Adverse Events **eTable 12.** Summary of Systemic Adverse Events **eFigure.** Log-log Survival Plot for Primary Outcome
eReferences

Supplement 3.
Data Sharing Statement
