# Supplementary material for: Early vs Deferred Non–Messenger RNA COVID-19 Vaccination Among Chinese Patients With a History of Inactive Uveitis: A Randomized Clinical Trial
Source: JAMA Netw Open. 2023 Feb 14;6(2):e2255804. doi: 10.1001/jamanetworkopen.2022.55804 (PMC9929699; doi:10.1001/jamanetworkopen.2022.55804)
Supplement: Supplement 1. — Trial Protocol [file jamanetwopen-e2255804-s001.pdf]

A Pragmatic, Randomized Clinical Trial of Coronavirus (COVID-19) Vaccinations in Uveitis

Study Protocol

Version Number: 2.0

The First Affiliated Hospital of Chongqing Medical University,

Chongqing Key Laboratory of Ophthalmology and Chongqing Eye Institute,

Chongqing, China

Protocol Title: A Pragmatic, Randomized Clinical Trial of Coronavirus (COVID-19) Vaccinations in Uveitis

Version Number: 2.0

Protocol Date: September 1st, 2021

Site: Single center (The First Affiliated Hospital of Chongqing Medical University)

Grant: Chongqing Key Laboratory of Ophthalmology (CSTC, 2008CA5003) and National Natural Science Foundation of China

Chief Investigator: Peizeng Yang, MD., PhD

Chief Investigator's Correspondence: Department of Ophthalmology, The First Affiliated Hospital of Chongqing Medical University, Chongqing 400016, China; Tel: [redacted]; E-mail: peizengycmu@126.com

Back-up 24 Hour Global Emergency Contact Call: [redacted]

The back-up 24 hour global emergency contact call should only be used if you are not able to reach the Clinical Research Physician(s) or Study Coordinator(s) for emergency calls.

Document Revision History

Version

1. Protocol 1.0, July 1st, 2021

2. Protocol 2.0, September 1st, 2021

Revision in 4.1 Inclusion Criteria

Given the fact that a coronavirus disease 2019 (COVID-19) vaccine is now available to children ages 12 through 17 in China, "Aged 18 years or older" changed to "Aged 12 years or older".

Revision in 5.1 Entering Screening

"A signed informed consent will be obtained from the subject or their legally authorized representative before any study-related procedures are undertaken" changed to "A signed informed consent, or proxy consent in the case of minors (aged < 18 years), will be obtained from the subjects, or parents/legal guardian of minors, or their legally authorized representative before any study-related procedures are undertaken. Under appropriate circumstances, minors should approach to provide assent by a member of the research team experienced with minors. The minor should personally write their name and date the assent form, which is then signed by the parent/legal representative and the researcher and copies retained/disseminated as for consent forms"

Revision in 5.6 Follow-Up

"The vaccination records of all participants, including doses, times and vaccine types, will be verified at the Month 3 visit by seeking data from the Health Code System which has a linkage to databases of routinely collected individual-level

vaccination information held by the Local Health Administrative Committee" added.

Revision in 10 Ethical Considerations

"Subjects must provide informed consent to participate in the trial" changed to "Consent from the patient, or proxy consent in the case of minors, should be obtained by a designated member of the research team prior to their participation in the trial, after a full explanation has been given of the intervention options".

CONTENTS

CONTENTS 5

ABBREVIATIONS 8

1 Introduction 9

1.1 Background 9

1.2 Benefits and Risks 11

2 Objectives 13

2.1 Trial Objectives 13

2.2 Primary Objective 13

2.3 Secondary Objectives 13

2.4 Safety Objectives 13

3 Study Design 14

3.1 Overall Design 14

3.2 Study Schematic 14

3.3 Study Timeline 15

4 Eligibility Criteria 16

4.1 Inclusion Criteria 16

4.2 Exclusion Criteria 16

4.3 Re-screening of Patients 17

5 Study Procedures 18

5.1 Entering Screening 18

5.2 Eligibility Evaluation 18

5.3 Randomization Assignment and Masking 18

5.4 Baseline Assessments 19

5.5 Interventions 20

5.6 Follow-Up 20

5.7 Unscheduled Visits 27

5.8 Withdrawn and Lost to Follow-up 27

5.9 Special Ocular Examinations 28

5.10 Study Schedule Overview 29

6 Outcome Assessments 31

6.1 Adjudication Committee 31

6.2 Primary Outcome 31

6.3 Secondary Outcomes 31

6.4 Systemic Outcomes 33

6.5 Pre-specified Subgroup Analyses 33

7 Adverse Events 34

7.1 Adverse Event Reporting 34

7.2 Serious Adverse Events 34

7.3 Severity Assessments 35

7.4 Causality Assessments 35

7.5 Uveitis-Related Events 35

7.6 Pregnancy 36

7.7 Management 36

8 Data Collection and Management 38

8.1 Confidentiality 38

8.2 Source and Data Collection 38

8.3 Quality Assurance 38

8.4 Database Lock 39

8.5 Archiving 39

9 Statistical Considerations 40

9.1 Sample Size Calculation 40

9.2 Data Analyses 40

9.3 Interim Analysis 41

10 Ethical Considerations 42

REFERENCES 43

Appendix I 44

Appendix II 45

ABBREVIATIONS

AE	Adverse event
BCVA	Best corrected visual acuity
CFDA	China Food and Drug Administration
COVID-19	Coronavirus Disease 2019
CRF	Case report form
FFA	Fluorescein fundus angiography
NEI	National Eye Institute
OCT	Optical coherence tomography
SUN	Standardization of Uveitis Nomenclature Working Group

1 Introduction

1.1 Background

Why do we need a vaccine for COVID-19?

As the Coronavirus Disease 2019 (COVID-19) continues to affect and kill thousands of people around the world, politicians, health professionals and the public want to know how to get out of the blockade and begin to return to normal life. Without a vaccine against SARS-COV-2, the coronavirus that causes COVID-19, there is always a risk of new disease outbreaks. While rigorous testing, contact tracking, and isolation procedures will help control the spread of COVID-19, the only way to significantly reduce the threat is to immunize a large enough population against the virus (often known as herd immunity) so that the population cannot transmit it.[1,2] To achieve this, it is estimated that about 60% of the population to be immune to the disease[1,2] Everyone 18 years of age and older is now eligible to get a COVID-19 vaccination. Widespread vaccination is a critical tool to help stop the pandemic.

Do people with underlying medical conditions need a vaccine for COVID-19?

Adults of any age with certain underlying diseases are at increased risk of developing serious diseases due to the virus that causes COVID-19. The COVID-19 vaccine is recommended and can be administrated in most people with underlying diseases. People with autoimmune conditions may receive a COVID-19 vaccine. However, we should be aware that no data are currently available for this population. People from this group were eligible for enrollment in some of the clinical trials. However, evidence for the timing of COVID-19 vaccination in patients with autoimmune disease is still lacking.

What is uveitis?

Uveitis is one of several vision-threatening diseases. Originally, uveitis refers to a

collection of diseases characterized by intraocular inflammation that mainly affect the uvea, consisting of the iris, ciliary body and choroid. Uveitis also encompasses a range of entities involving inflammation of adjoining intraocular structures including the retina, vitreous and optic nerve.[3] According to the major site of inflammation, uveitis can be anatomically classified into anterior uveitis (inflammation of the iris and anterior chamber), intermediate uveitis (inflammation of the ciliary body and vitreous body), posterior uveitis (inflammation of the vitreous body, retina, choroid or even the optic disk) and panuveitis (a diffuse inflammation from the anterior through the posterior segment of the eye).[4]

Uveitis can also be classified into two categories, infectious or non-infectious, and the standard treatments for the two categories are largely different. Infectious uveitis can be caused by a localized or systemic infection with a definite pathogens, such as cytomegalovirus, herpes simplex virus (HSV), *Toxoplasma gondii, Treponema pallidum* and *Mycobacterium tuberculosis*, which requires anti-infection therapy.[5] Non-infectious uveitis can be associated with systemic diseases including spondyloarthritis, Behçet's disease, Vogt-Koyanagi-Harada disease (VKH) and sarcoidosis. In addition, non-infectious uveitis may be confined to the eye with no apparent associated systemic diseases, such as sympathetic ophthalmia, birdshot chorioretinopathy, serpiginous choroiditis, or multiple evanescent white dot syndrome.[6] Most non-infectious uveitis entities are presumed to be an immune-related disorder, usually termed as autoinflammatory or autoimmune disease, which means a self-directed pathological process. In clinical practice, these entities are primarily treated with immunosuppressive agents to suppress the autoinflammatory or autoimmune responses.[7]

What is the concern on vaccination in uveitis?

Having uveitis is currently not a contraindication (a medical reason to avoid) the vaccine. In fact, getting the vaccine when having uveitis may be especially important,

given the disease may confer a higher risk for COVID-19 or severe outcomes. However, it is possible that individuals could have an immune response to the COVID-19 vaccine. It remains unclear whether this immune response may significantly impact on the outcome of patients with uveitis. Furthermore, uveitis may also be a possible adverse side effect of some other vaccines.[8-10] Given the fact that patients with uveitis are on immunosuppressants like steroids, methotrexate, or biologics, they may be on different disease status (active, inactive or remission), and they have different causes (infectious or non-infectious), it is unknown about the best timing of COVID-19 vaccination which can cause minimal adverse events on uveitis patients.

What is the key question of our study?

In this study, we aim to initiate a pragmatic randomized trial to investigate whether there is significant difference in outcomes of patients with an inactive uveitis (active uveitis is considered not eligible for COVID-19 vaccination) between prompt initiation of COVID-19 vaccination and deferred initiation of COVID-19 vaccination until uveitis remission.

1.2 Benefits and Risks

The risks to participants are minimal with randomizing COVID-19 vaccination recommendations as part of clinical care, as it is simply testing two different vaccination strategies. The primary potential risk will be uveitis relapse. However, uveitis relapse is common and can be properly treated, and relapse related visual loss can be adequately and timely prevented provided a close monitoring. To minimize potential risk, we will implement best medical judgment and treatment if any study endpoint event occurs to assure the safety of participants.

The potential for public health benefit from our aims to randomize COVID-19 vaccination recommendations is of importance, potentially to the benefit of future

COVID-19 vaccination recommendation and decision in patients with uveitis. This study would provide direct evidence and timely data to guide physician to give proper recommendations on COVID-19 vaccination for patients with uveitis.

2 Objectives

2.1 Trial Objectives

The objective is to test the difference in ocular and systemic outcomes between prompt initiation of COVID-19 vaccination and deferred initiation of COVID-19 vaccination in patients with uveitis.

2.2 Primary Objective

To compare the time to symptomatic uveitis worsening between prompt and deferred vaccination strategies for patients with uveitis.

2.3 Secondary Objectives

To compare vaccine accessibility, patient compliance, clinically-confirmed uveitis worsening, visual outcomes, treatment interruptions and initiation of step-up treatment, hospitalization, laboratory-confirmed COVID-19 between prompt and deferred vaccination strategies for patients with uveitis.

2.4 Safety Objectives

To evaluate the systemic adverse events of the prompt versus deferred vaccination strategies for patients with uveitis.

3 Study Design

3.1 Overall Design

This is a pragmatic, randomized clinical trial to test the difference in ocular and systemic outcomes between prompt initiation of COVID-19 vaccination and deferred initiation of COVID-19 vaccination in uveitis patients who have not yet been vaccinated and whose ocular disease maintains inactive but has not yet achieved complete remission.

Approximately 1314 eligible subjects will be randomized 1:1 (657 subjects per group) to receive one of two vaccination strategy recommendations. In the prompt vaccination arm, subjects will be recommended to get a prompt initiation of COVID-19 vaccination. In the deferred vaccination arm, subjects will be recommended to get the vaccination after uveitis remission (inactive disease for 3 months after discontinuing all treatments for eye disease). All participants will be followed up for 3 months since randomization. The primary outcome will be the difference in time to symptomatic uveitis worsening.

3.2 Study Schematic

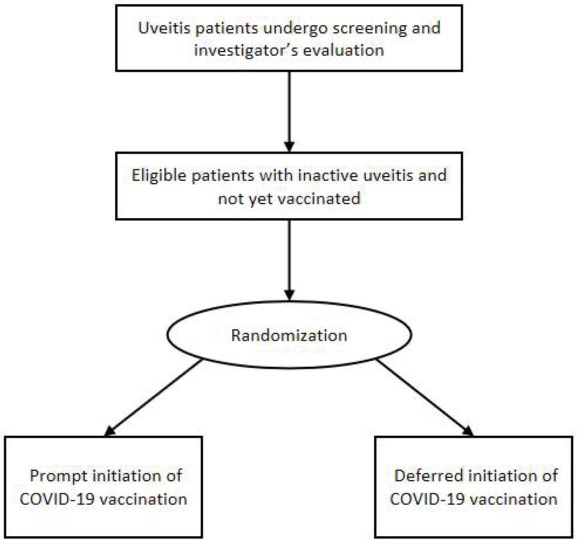

3.3 Study Timeline

Expected time for trial start: July 2021

Expected completion time of enrollment: October 2021

Expected time for end of trial: January 2022

The end of trial refers to the date of the last visit of the last subject to complete the study, or the date of receipt of the last data point from the last subject that is required for primary, secondary and/or exploratory analysis, as pre-specified in the protocol and/or the Statistical Analysis Plan, whichever is the later date.

Expected completion time of primary analysis: May 2022

4 Eligibility Criteria

An important goal of pragmatic randomized trials is to provide relevant and generalizable information to guide clinical decision-making. As such, the inclusion and exclusion criteria of pragmatic trials is often more broad than those of traditional randomized controlled trials.

4.1 Inclusion Criteria

A subject will be eligible for study participation if she/he meets all of the following inclusion criteria:

a. Aged 12 years or older

b. Not yet vaccinated prior to the Screening.

c. Diagnosed with any forms of uveitis in any eye.

d. An **inactive disease** status (all the following criteria are met):

   (1) Anterior chamber cells of 0.5+ or less (See SUN criteria[11] in Appendix I);
   (2) Vitreous haze of 0.5+ or less (See NEI/SUN criteria[12] in Appendix II);
   (3) No active inflammatory choroidal or retinal vascular lesions.

4.2 Exclusion Criteria

The presence of any of the following will exclude a subject from the study enrollment:

a. Complete uveitis remission: Inactive disease for 3 months after discontinuing all treatments for eye disease.

b. Any known vaccine contraindications according to manufactures' instructions:

   (1) Individuals with a history of anaphylaxis to any component of the vaccine;
   (2) Individuals with a body temperature over 38.5°C;
   (3) Pregnancy.

c. Any other condition which, in the opinion of the Investigator, would put the subject at risk by participation in the protocol.

4.3 Re-screening of Patients

Individuals that do not meet the above eligibility criteria may be re-screened a minimum of 2 weeks after the last screening visit.

5 Study Procedures

5.1 Entering Screening

Procedures for obtaining consent include explaining the patient's disease, prognosis, and options, introducing the aims and methods of this trial, discussing the risks and benefits of participation and addressing the patient's questions and concerns. Patients will be given at least 24 hours to consider taking part. The subject is assured that participation in this study is voluntary and he/she can withdraw at any time, without giving a reason. A signed informed consent, or proxy consent in the case of minors (aged < 18 years), will be obtained from the subjects, or parents/legal guardian of minors, or their legally authorized representative before any study-related procedures are undertaken. Under appropriate circumstances, minors should approach to provide assent by a member of the research team experienced with minors. The minor should personally write their name and date the assent form, which is then signed by the parent/legal representative and the researcher and copies retained/disseminated as for consent forms.

5.2 Eligibility Evaluation

The eligibility evaluation will be performed at the Screening. For detailed inclusion/exclusion criteria see Section 4.

The Investigator will perform slit-lamp biomicroscopy, ophthalmoscopy and visual acuity on each candidate during the Screening. Other auxiliary examinations including fundus photography, optical coherence tomography (OCT) and fundus fluorescence angiography (FFA), may be performed at the Investigators' discretion according to the clinical needs to evaluate patient's ocular condition, and examination results or reports will be recorded on medical notes.

5.3 Randomization Assignment and Masking

After eligibility has been determined, assignment of a randomization takes place at the Baseline visit. Only subjects who meet all of the inclusion criteria and none of the exclusion criteria are eligible for randomization into the treatment phase. Randomization will be designed to yield expected assignment ratio of 1:1 to the prompt vaccination strategy and the deferred vaccination strategy. Randomization list will have been generated by a Statistician using a computer and will be properly kept by a Designated Study Coordinator. Both this Statistician and the Designated Study Coordinator will have no involvement in other parts of the trial. The Investigator who is designated as the Primary Treating Ophthalmologist and is responsible for the management of the subject in this trial will telephone the Designated Study Coordinator to obtain patient assignment information. Throughout the trial, the randomization assignment will not be masked to the Primary Treating Ophthalmologist, but the Primary Treating Ophthalmologist will not have direct access to the randomization list. The randomization assignment will be masked to an adjudication committee who will independently determine the study endpoint events according to medical data, records and examination reports written by the Primary Treating Ophthalmologist, the attending doctor or the examining doctor. **These medical notes and data will be collected by a group of Study Coordinators who mask any randomization assignment information and submit these materials online to the adjudication committee.**

5.4 Baseline Assessments

The Baseline visit becomes time-point zero from which all other visit dates are calculated. The Screening and the Baseline can be performed on the same day. Considering the pragmatic study design, to ensure a rapid enrollment and data collection for a broad range of participants, baseline clinical characteristics and medical history can be either reported by patients or retrieved by means of a study site-specific query of the health record. Pre-specific baseline data include the date of

birth, gender, ethnic group, height, weight, presence of hypertension, diabetes, coronary heart disease, malignancies, type of uveitis (anterior, intermediate, posterior or panuveitis), specific uveitis diagnosis, affected eye (right, left or both), duration of uveitis, number of flares in the past 12 month, best corrected visual acuity logMAR score, and intraocular pressure.

5.5 Interventions

Interventions will take place at the Baseline visit. The Primary Treating Ophthalmologist will provide the recommendation of COVID-19 vaccination to the subject according to randomization assignment. For those randomized to the prompt vaccination, subjects will be recommended to go to vaccination site and get COVID-19 vaccination as soon as possible. Our ophthalmologic clinic does not provide the vaccination service. We will only give the recommendation to patients. For those randomized to the deferred vaccination, subjects will be recommended to get COVID-19 vaccination after the remission of uveitis. **Remission is defined as an inactive disease for 3 months after discontinuing all treatments for eye disease.** At baseline, all patients will also be educated to immediately contact us by phone call once they experience any discomfort or have any new ocular symptoms of eye redness, eye pain, decreased vision, light sensitivity or fark, floating spots. We will ask questions as prespecified in **5.6 Follow-up** on the phone call.

5.6 Follow-Up

Routine follow-up encounters will then occur every month. Participants will complete trial visits primarily by telephone calls from the call center. However, all participants will need to complete an in-person clinic visit on the end of three months since baseline.

On phone call, patients will be asked to answer the following predetermined questions according to their randomization assignment. **Module 1** will be subjected to those in

prompt vaccination group, and **Module 2** will be subjected to those in deferred vaccination group.

Module 1

Question 1: Do you think you have had a uveitis worsening or disease recurrence (relapse) in the past month?

Yes, no or uncertain. If yes, specify which eye and duration.

Question 2: Have you ever experienced newly occurred eye redness in the past month?

Yes, no or uncertain. If yes, specify which eye and duration.

Question 3: Have you ever experienced newly occurred eye pain in the past month?

Yes, no or uncertain. If yes, specify which eye and duration.

Question 4: Have you ever experienced new blurred vision or decreased vision in the past month?

Yes, no or uncertain. If yes, specify which eye and duration.

Question 5: Have you ever experienced newly occurred light sensitivity in the past month?

Yes, no or uncertain. If yes, specify which eye and duration.

Question 6: Have you ever had newly occurred dark, floating spots in your field of vision (floaters, specks or moving clouds in your vision) in the past month?

Yes, no or uncertain. If yes, specify which eye and duration.

Question 7: Have you ever experienced other ocular or systemic symptoms in the past month?

Yes, no or uncertain. If yes, specify where and duration.

Question 8: Have you already been received any dose of COVID-19 vaccination?

Yes or no. If no, jump to Question 14.

Question 9: Which COVID-19 vaccine did you get?

Sinovac (CoronaVac), Sinopharm, ZFSW Biologics Company, others (specifically), or uncertain.

Question 10: How many doses of COVID-19 vaccines have you already been received?

One, two, three or more (specific numbers).

Question 11: When did you get each dose of COVID-19 vaccines?

Participants will be asked to provide the specific date as far as possible.

Question 12: Have you finished all required doses of COVID-19 vaccines?

Yes or no. If the answer is no, continue on Question 13, otherwise finished.

Question 13: When do you plan to get the next vaccine?

Participants will be asked to provide the specific date as far as possible. If participants answer they no longer intend to get the next vaccine, specific reasons should be provided. Questions finished.

Question 14: Why did not you get vaccinated?

Patients will be asked to provide the reason for not having COVID-19 vaccination. Not willing to get vaccination, willing to get vaccination but having not yet gone to the vaccination site (specific reasons for not yet gone to the vaccination site required), having gone to the vaccination site but refused due to the lack of vaccines, having gone to the vaccination site but refused due to contraindication, other reasons (specifically). After completing this question, all participants will be encouraged to go to get vaccination as soon as possible on the phone, although they might have shown

their unwillingness to get one or they might have tried but failed.

At the end of phone call, if the patient answer yes to any of questions 1 to 7 in the Module 1, the patient will be instructed to come back to study site to complete in-person clinic visit for further evaluation and examination. Alternatively, the patient will also be allowed to go to local hospitals for evaluation and examination, but the patient need to send back the copies of all medical notes, records, laboratory or auxiliary examination reports from the local hospitals. **Participants will receive best medical judgment and proper treatment for worsening of the ocular or systemic conditions, including but not limited to step-up treatment, treatment interruptions even hospitalization. Nasopharyngeal swabs for RT-PCR testing of SARS-COV-2 will be performed on cases of hospitalization.** No matter where the evaluation is completed, patients will be instructed to undergo three mandatory ocular assessments, including **best-corrected visual acuity testing, slit-lamp biomicroscopy and ophthalmoscopy**. Mandatory materials send back from local hospitals include the medical record written by the attending doctor with signature and the prescription of that clinic visit. An adjudication committee who are unaware of group assignment will independently determine the endpoint according to these medical records from our study site or the local hospitals. **If clinically-confirmed uveitis worsening occurs, participants will be recommended to postpone subsequent vaccination until uveitis remission.**

Module 2

Question 1: Do you think you have had a uveitis worsening or disease recurrence (relapse) in the past month?

Yes, no or uncertain. If yes, specify which eye and duration.

Question 2: Have you ever experienced newly occurred eye redness in the past month?

Yes, no or uncertain. If yes, specify which eye and duration.

Question 3: Have you ever experienced newly occurred eye pain in the past month?

Yes, no or uncertain. If yes, specify which eye and duration.

Question 4: Have you ever experienced new blurred vision or decreased vision in the past month?

Yes, no or uncertain. If yes, specify which eye and duration.

Question 5: Have you ever experienced newly occurred light sensitivity in the past month?

Yes, no or uncertain. If yes, specify which eye and duration.

Question 6: Have you ever had newly occurred dark, floating spots in your field of vision (floaters, specks or moving clouds in your vision) in the past month?

Yes, no or uncertain. If yes, specify which eye and duration.

Question 7: Have you ever experienced other ocular or systemic symptoms in the past month?

Yes, no or uncertain. If yes, specify where and duration.

Question 8: Have you been disease-free and discontinuing uveitis treatment for 3 months?

Yes or no. If yes, jump to Question 15.

Question 9: Have you already been received any dose of COVID-19 vaccination?

Yes or no. If no, participants will be recommended to keep away from getting vaccination at present and the conversation will be finished, otherwise continue on Question 10.

Question 10: Which COVID-19 vaccine did you get?

Sinovac (CoronaVac), Sinopharm, ZFSW Biologics Company, others (specifically), or uncertain.

Question 11: How many doses of COVID-19 vaccines have you already been received?

One, two, three or more (specific numbers).

Question 12: When did you get each dose of COVID-19 vaccines?

Participants will be asked to provide the specific date as far as possible.

Question 13: Have you finished all required doses of COVID-19 vaccines?

Yes or no. If the answer is no, continue on Question 14, otherwise finished.

Question 14: When do you plan to get the next vaccine?

Participants will be asked to provide their plan on specific date as far as possible. If participants answer they no longer intend to get the next vaccine, specific reasons should be provided. Questions finished.

Question 15: Have you already been received any dose of COVID-19 vaccination?

Yes or no. If yes, back to Question 10. If no, jump to Question 16.

Question 16: Why did not you get vaccinated?

Patients will be asked to provide the reason for not having COVID-19 vaccination. Not willing to get vaccination, willing to get vaccination but having not yet gone to the vaccination site (specific reasons for not going to the vaccination site required), having gone to the vaccination site but refused due to the lack of vaccines, having gone to the vaccination site but refused due to contraindication, other reasons (specifically). After completing this question, all participants will be encouraged to go to get vaccination as soon as possible on the phone, although they might have shown their unwillingness to get one.

At the end of phone call, if the patient answer yes to any of questions 1 to 7 in the Module 2, the patient will be instructed to come back to study site to complete in-person clinic visit for further evaluation and examination. Alternatively, the patient will also be allowed to go to local hospitals for evaluation and examination, but the patient need to send back the copies of all medical notes, records, laboratory or auxiliary examination reports from the local hospitals. **Participants will receive best medical judgment and proper treatment for worsening of the ocular or systemic conditions, including but not limited to step-up treatment, treatment interruptions even hospitalization. Nasopharyngeal swabs for RT-PCR testing of SARS-COV-2 will be performed on cases of hospitalization.** No matter where the evaluation is completed, patients will be instructed to undergo three mandatory ocular assessments, including **best-corrected visual acuity testing, slit-lamp biomicroscopy and ophthalmoscopy**. Mandatory materials send back from local hospitals include the medical record written by the attending doctor with signature and the prescription of that clinic visit. An adjudication committee who are unaware of group assignment will independently determine the endpoint according to these medical records from our study site or the local hospitals. **If clinically-confirmed uveitis worsening occurs, participants will be recommended to postpone subsequent vaccination until uveitis remission. If a patient assigned to the deferred vaccination group is vaccinated without remission of his/her ocular disease, we will no longer provide any recommendations on vaccination and implement assigned intervention strategies, this patient will only be observed later.**

The vaccination records of all participants, including doses, times and vaccine types, will be verified at the Month 3 visit by seeking data from the Health Code System which has a linkage to databases of routinely collected individual-level vaccination information held by the Local Health Administrative Committee.

5.7 Unscheduled Visits

Participants will encounter at the clinic and have unscheduled visit for any reasons. They will be asked to answer questions in Module 1 or Module 2 according to their assignment. They will also undergo three mandatory assessments, including **best-corrected visual acuity testing, slit-lamp biomicroscopy and ophthalmoscopy,** and other examinations at investigator's discretion. For those with disease worsening, participants will receive best medical judgment and proper treatment for worsening of the ocular or systemic conditions.

5.8 Withdrawn and Lost to Follow-up

Subjects have the right to withdraw from the trial at any time and for any reason, without providing a reason. The investigator has the right to withdraw participants from the study and abandon assigned intervention strategies in the event of inter-current illness, adverse events, protocol violations or other reasons.

If the subject withdraws from the trial, efforts will be made to continue to obtain follow-up data, with the permission of the subject. They will be given appropriate treatment, but will not continue with scheduled study follow-ups unless they allow it.

Subjects will also be considered to have been withdrawn if they declare they are no longer interested in further participation, or have died. If the death is considered to be related to the vaccines, the subject will be declared serious adverse event, rather than withdrawn.

Missing a phone call or visit does not necessarily mean that a patient has been withdrawn from the study. For these subjects, efforts will be made to contact them and bring them back as soon as possible.

For those subjects fail to response the contact or to attend study visits without stating

an intention to withdraw from the study, the Investigator should contact the subject through telephone calls as soon as possible. The investigator will attempt to contact them for at least three times at each scheduled visit. If contact cannot be established up to the end of the trial, the subject should be considered as “lost to follow-up” on the date of last contact.

5.9 Special Ocular Examinations

Visual acuity testing

With the appropriate corrective lenses based on that subject’s refraction, the best corrected visual acuity (BCVA) will be measured using a Logarithmic visual acuity chart or according to local routine practice. The subject’s presenting visual acuity without refraction will also be evaluated. Visual acuity will be obtained prior to pupil dilation. Data will be transformed into LogMAR score.

Tonometry

Intraocular pressure for both eyes will be measured using a non-contact tonometry. Tonometry should be performed prior to pupil dilation.

Slit-lamp biomicroscopy

A standard ophthalmic examination using slit lamp biomicroscopy will be performed in both eyes by the unmasked Primary Treating Ophthalmologist. The Anterior chamber cell count will be measured during the examination according to the SUN criteria (see Appendix I), which should be performed prior to pupil dilation.

Ophthalmoscopy

Ophthalmoscopy will be performed by the Primary Treating Ophthalmologist under the dilation of the pupil to determine both vitreous haze grading and the absence/presence of active chorioretinal and/or retinal vascular lesions. Grading of vitreous haze will be based on the publication from the National Eye Institute (NEI)

which has also been adapted by the SUN working group (see Appendix II).

Fundus photography

Fundus photography will be performed to obtain evidence of the absence/presence of active chorioretinal and/or retinal vascular lesions.

Optical coherence tomography (OCT)

Optical coherence tomography will be used to determine the absence/presence of retinal lesions and macular alternations.

Fundus fluorescence angiography (FFA)

Fundus fluorescence angiography will be performed to determine the absence/presence of active chorioretinal and/or retinal vascular lesions.

5.10 Study Schedule Overview

	**Screening/Baseline**	**Month 1 (phone call)**	**Month 2 (phone call)**	**Month 3 (in person)**	**End trial month after Month 3 (phone call)**	**Post-symptom visit (our study site or local hospital)**	**Unscheduled visit (in person)**
Visit window (day)	0	±7	±7	±14	±7	-	-
Written informed consent	×						
Inclusion/exclusion criteria	×						
Randomization	×						
General information and medical history	×						
Answer to Questions (Module 1 or Module 2)		×	×	×*	×		×
Visual acuity testing	×			×		×	×
Slit-lamp biomicroscopy	×			×		×	×
Ophthalmoscopy	×			×		×	×
Any other necessary	×			×		×	×

	Screening/Baseline	Month 1 (phone call)	Month 2 (phone call)	Month 3 (in person)	End trial month after Month 3 (phone call)	Post-symptom visit (our study site or local hospital)	Unscheduled visit (in person)
Visit window (day)	0	±7	±7	±14	±7	-	-
auxiliary examination at discretion							
Treatment, prescription and record	×			×		×	×
Adverse event evaluation		×	×	×	×	×	×

* can be assessed by phone call.

6 Outcome Assessments

6.1 Adjudication Committee

An adjudication committee who are unaware of group assignment will independently determine each endpoint event.

6.2 Primary Outcome

The primary outcome will be the time to symptomatic worsening over the course of 3 months of follow-up.

Symptomatic worsening is defined if one of following newly onset symptoms occurs in at least one eye lasting for at least 2 days: eye redness, eye pain, decreased vision, light sensitivity or floaters.

6.3 Secondary Outcomes

a. Time to clinically-confirmed uveitis worsening.

Clinically-confirmed uveitis worsening is defined if any of the following criteria are met in at least one eye: new active, inflammatory chorioretinal or retinal vascular lesions (determined by clinical examination such as ophthalmoscopy or ancillary testing such as fundus photography and fluorescein angiography); worsening of best corrected visual acuity by 0.3 LogMAR score or more; a two-step increase in anterior chamber cell grade relative to baseline; or a two-step increase in vitreous haze grade relative to baseline. *A 2-step increase is represented by a change of grade 0 to grade 2+; or grade 0.5+ to grade 3+.*

b. Proportion of each reason for symptomatic uveitis worsening.

c. Proportion of symptomatic uveitis worsening.

d. Proportion of each reason for clinically-confirmed uveitis worsening.

e. Time to initiation of step-up treatment.

Step-up treatment is defined if any of the following criteria are met: addition of another systemic immunosuppressive agent (or biologics), switching to another systemic immunosuppressive agent (or biologics), or increasing doses of any currently systemically administrated drugs.

f. Time to treatment interruptions.

Treatment interruptions are defined if any of the following criteria are met: decreasing doses of or discontinuing any currently systemically administrated drugs due to adverse events rather than disease improvement.

g. Time to hospitalization for any reasons.

h. Time to hospitalization for ocular disease.

i. Time to laboratory-confirmed COVID-19.

j. Patient adherence, defined as proportion correctly vaccinated for at least one dose or maintaining not vaccinated per protocol throughout the trial.

k. Proportion of vaccinated type and dose.

l. Proportion of uveitis remission throughout the trial.

Uveitis remission is defined as an inactive disease for 3 months after discontinuing all treatments for eye disease.

m. Vaccine inaccessibility, defined as proportion having gone to the vaccination site but refused due to the lack of vaccines, whereby this situation continues until the end of trial.

n. Reasons for not yet vaccinated until the end of trial among those who should have been vaccinated per protocol.

o. Change in anterior chamber cells from the Baseline to the end of three months.

p. Change in vitreous haze from the Baseline to the end of three months.

q. Change in best corrected visual acuity LogMAR score from the Baseline to the end of three months.

6.4 Systemic Outcomes

The systemic outcome will be systemic adverse events (apart from uveitis related events).

6.5 Pre-specified Subgroup Analyses

To determine differences between assignment groups in outcomes across subgroup variable defined by uveitis anatomical subtypes, etiology diagnosis classification, history of uveitis flare, age and sex.

7 Adverse Events

7.1 Adverse Event Reporting

An adverse event (AE) is defined as any unfavorable medical occurrence in a subject who has ever received study intervention, regardless of a causal relationship with this intervention. Any worsening of a pre-existing condition or illness should be considered an adverse event. Worsening in severity of a reported adverse event should also be reported as a new adverse event. An elective surgery/procedure scheduled to occur during a study will not be considered an adverse event if the surgery/procedure is being performed for a pre-existing condition and the surgery/procedure has been pre-planned prior to study entry. However, if the pre-existing condition deteriorates unexpectedly during the study (e.g., surgery performed earlier than planned), then, the deterioration of the condition for which the elective surgery/procedure is being done will be considered an adverse event. For adverse events to be considered intermittent, the events must be of similar nature and severity.

The Investigator will monitor each subject for clinical and laboratory evidence of adverse events throughout the study. The Investigator will assess and record any adverse event in detail including the date of onset, event diagnosis (if known) or sign/symptom, severity, time course, duration and outcome, relationship of the adverse event to study intervention and any actions taken. All these information on non-serious and serious adverse events will be recorded at each study visit.

7.2 Serious Adverse Events

Serious adverse events (SAE) should be reported to Chief Investigator and Ethics Committee within 24 hours of the Investigator being made aware of the SAE. Serious adverse events include any medical occurrence that results in the following outcomes:

Death.

Life-threatening experience.

Non-elective surgery or hospitalization for any reason.

Congenital anomaly or birth defect.

Persistent or significant disability or incapacity.

Any other important medical events, not immediately life-threatening or resulting in death or hospitalization but requiring medical or surgical intervention to prevent serious outcome.

7.3 Severity Assessments

The severity of adverse event can be assessed according to the following criteria:

Mild: The adverse event does not interfere with the volunteer's daily routine, and does not require intervention; it causes slight discomfort.

Moderate: The adverse event interferes with some aspects of the volunteer's routine, or requires intervention, but is not damaging to health; it causes moderate discomfort.

Severe: The adverse event results in alteration, discomfort or disability which is clearly damaging to health.

7.4 Causality Assessments

The Investigator should assess the relationship of the adverse event with the use of vaccines by reporting according to the three-level classification of "associated", "not associated", and "inability to determine". Causality assessments will be required for study drugs as well as other adjunctive treatments.

7.5 Uveitis-Related Events

The following events are known complications related to the condition being treated and will be classified as uveitis-related events. These events will be analyzed

separately from other adverse events in the final study report. Notably, the following events may not be a complete list of all potential uveitis-related events. The Investigators must determine if a specific event is uveitis-related.

Loss of transparency of the cornea.

Band keratopathy.

Synechiae.

Cataracts.

Glaucoma/increased intraocular pressure.

Vitreous hemorrhage.

Macular edema.

Retinal detachment.

Epiretinal membrane.

Vitreo-macular traction.

Retinal ischemia.

Vision loss.

Hypotony.

7.6 Pregnancy

Subjects who become pregnant during the study period must be discontinued. Pregnancy in a study subject is not considered as an adverse event. However, the medical outcome of an elective or spontaneous abortion, stillbirth or congenital anomaly is considered as a serious adverse event.

7.7 Management

For the purpose of medical management, all adverse events and laboratory abnormalities that occur during the study must be evaluated by the Investigator and treated according to the best medical judgment of their clinician. The occurrence of adverse events does not necessarily mean the time for the subject to exit the study. But, a serious adverse event will absolutely result in the discontinuation of the study immediately. For those developing non-serious adverse events, administration of other adjunctive treatments or even stopping the assigned intervention may be used.

8 Data Collection and Management

8.1 Confidentiality

Participants will be identified via a unique ID. Identifiable information will not be stored in the eCRF and will not leave the site. Any participant contact information will be stored within the site on password protected computers or within secured locations with limited access. All study data and site files will be kept at site in a secure location with restricted access.

8.2 Source and Data Collection

The study will employ an eCRF, whereby data will be managed via this system. Source data worksheets will be used for each patient and data will be entered onto the eCRF database. Source data worksheets will be reconciled at the end of the trial with the patients' medical notes in the study site. During the trial, critical clinical information will be written in the medical notes to ensure informed medical decisions can be made in the absence of the study team. Trial related clinical letters will be copied to the medical notes during the trial. The Chief Investigator will provide a signature for CRF once all queries are resolved and immediately prior to database lock.

It will be the responsibility of the Chief Investigator and his team to ensure the accuracy of all data entered in the worksheets and the eCRF are in accordance with Good Clinical Practice. The Principal Investigator will be responsible for ensuring that source data worksheets are filed in a suitably secure location to ensure source data verification can be undertaken throughout the study.

8.3 Quality Assurance

The study will incorporate a range of data management quality assurance functions as the trial progresses. The Trial Data Manager will provide study training, ongoing

study support and will conduct regular monitoring, checking source data for transcription errors. Any necessary alterations to entered data will be date and time stamped within the eCRF. Regular monitoring of study conduct and data collected will be performed to ensure the study is conducted in accordance with GCP.

8.4 Database Lock

Prior to database lock, the Trial Manager will review any outstanding warnings on the eCRF and resolve or close these as appropriate before database lock. Local study personnel should resolve any queries that arise promptly. Once all queries have been resolved no further changes will be made to the database unless specifically requested by the Study Office in response to the Statistician's data checks. The study Chief Investigator will review all the data and provide electronic signature to verify that all the data are complete and correct. At this point, all data will be formally locked for analysis.

8.5 Archiving

Chief Investigator will be responsible for securely archiving local data generated, essential documents and source data in accordance with local requirements, but for at least 5 years from the end of the study.

9 Statistical Considerations

9.1 Sample Size Calculation

A two-sided log-rank test with an overall sample size of 1314 subjects (657 in the deferred vaccination group and 657 in the prompt vaccination group) achieves 80.0% power at a 0.050 significance level to detect a hazard ratio of 1.5 when the proportion surviving (no occurrence of symptomatic uveitis worsening) in the deferred vaccination group is 0.9000. The study lasts for 6 time periods of which subject accrual (entry) occurs in the first 3 time periods. The accrual pattern across time periods is uniform (all periods equal). The proportion dropping out of both groups is 0.1500, respectively.

9.2 Data Analyses

Analysis methods of each study outcome will be provided in a detailed statistical analysis plan.

Primary analyses will be on an Intention-to-treat (ITT) basis. According to the intention-to-treat principle (ITT), all patients who have been randomized regardless of their compliance with the study intervention and whose primary outcome is available will be included, which is one of the main data sets for the effectiveness evaluation of this study. Given the pragmatic nature of the trial and the expected gaps in data on patient compliance with randomized intervention, we will not consider the per-protocol (PP) analysis as the primary analysis.

The clinical and demographic characteristics of the participants will be summarized with descriptive statistics. Continuous variables will be described with their distribution range, mean, median, standard deviation, and interquartile range, as appropriate. Categorical variables will be summarized as numbers and the proportions.

The primary outcome will be the time to symptomatic uveitis worsening. For the primary outcome, the multiplicative limit method (Kaplan-Meier) is used for univariate survival analysis, the median time to symptomatic uveitis worsening and survival rate of each group are calculated, and the survival rate curve is compared by the Log-rank test.

For the systemic outcomes, the types, number, and incidence of all systemic adverse events during the follow-up period will be described in a tabular form.

9.3 Interim Analysis

This study does not plan to perform interim analysis.

10 Ethical Considerations

This clinical trial will be conducted in compliance with the approved protocol, the Helsinki Declaration, the Drug Clinical trial Management Code issued by the CFDA, and corresponding regulations.

Before the commencement of this experiment, the approval of the ethics committee must be obtained.

The Investigator is expected to take any immediate action required for the safety of any patient included in this study, even if this action represents a deviation from the protocol.

During the clinical study, any changes made to this trial protocol should be reported to the Ethics Committee and placed on record.

Subjects must provide informed consent to participate in the trial. Consent from the patient, or proxy consent in the case of minors, should be obtained by a designated member of the research team prior to their participation in the trial, after a full explanation has been given of the intervention options.

If the subject and his or her legal representative are illiterate, the informed consent process shall be attended by a witness, who shall sign the informed consent form after oral consent by the subject or his or her legitimate representative.

A copy of the informed consent form and the contact information for the researcher and the ethics committee must be provided to the patient.

The study site must be equipped with the necessary medical rescue equipment and first aid drugs for emergency issues.

REFERENCES

1. Fontanet A, Cauchemez S. COVID-19 herd immunity: where are we? Nat Rev Immunol 2020;20:583-4.

2. Randolph HE, Barreiro LB. Herd Immunity: Understanding COVID-19. Immunity 2020;52:737-41.

3. Krishna U, Ajanaku D, Denniston AK, Gkika T. Uveitis: a sight-threatening disease which can impact all systems. Postgrad Med J 2017;93:766-73.

4. Tsirouki T, Dastiridou A, Symeonidis C, et al. A focus on the epidemiology of uveitis. Ocul Immunol Inflamm 2018;26:2-16.

5. Deschenes J, Murray PI, Rao NA, Nussenblatt RB. International Uveitis Study Group (IUSG): clinical classification of uveitis. Ocul Immunol Inflamm 2008;16:1-2.

6. Hsu Y-R, Huang JC-C, Tao Y, et al. Noninfectious uveitis in the Asia–Pacific region. Eye 2019;33:66-77.

7. Jabs DA, Rosenbaum JT, Foster CS, et al. Guidelines for the use of immunosuppressive drugs in patients with ocular inflammatory disorders: recommendations of an expert panel. Am J Ophthalmol 2000;130:492-513.

8. Esmaeli-Gutstein B, Winkelman JZ. Uveitis associated with varicella virus vaccine. Am J Ophthalmol 1999;127:733-4.

9. Heydari-Kamjani M, Vante I, Uppal P, Demory Beckler M, Kesselman MM. Uveitis Sarcoidosis Presumably Initiated After Administration of Shingrix Vaccine: Cureus. 2019 Jun 17;11(6):e4920. doi: 10.7759/cureus.4920.; 2019.

10. Cunningham ET, Jr., Moorthy RS, Fraunfelder FW, Zierhut M. Vaccine-Associated Uveitis: Ocul Immunol Inflamm. 2019;27(4):517-520. doi: 10.1080/09273948.2019.1626188.; 2019.

11. Jabs D, Nussenblatt R, Rosenbaum J, Standardization of Uveitis Nomenclature (SUN) Working Group. Standardization of uveitis nomenclature for reporting clinical data. Results of the first international workshop. Am J Ophthalmol 2005;140:509-16.

12. Nussenblatt RB, Palestine AG, Chan CC, Roberge F. Standardization of vitreal inflammatory activity in intermediate and posterior uveitis. Ophthalmology 1985;92:467-71.

Appendix I

SUN Working Group Grading System for Anterior Chamber Cells

Grade	Number of cells in field [a]
0	<1
0.5+	1-5
1+	6-15
2+	16-25
3+	26-50
4+	>50

[a] Field size is a 1mm by 1mm slit lamp beam. Grading should be done with the highest magnification and illumination and conducted in a completely dark room, prior to dilation.

From Jabs DA et al. Standardization of uveitis nomenclature for reporting clinical data. Results of the First International Workshop. Am J Ophthalmol 2005;140(3):509-16.

Appendix II

National Eye Institute (NEI)/SUN Criteria for Grading Vitreous Haze

Grade	Description [a]
0	No evident vitreal haze
0.5+	Slight blurring of the optic disc margin because of the haze; normal striations and reflex of the nerve fiber layer cannot be visualized
1+	Permits a better definition of both the optic nerve head and the retinal vessels (compared to higher grades)
2+	Permits better visualization of the retinal vessels (compared to higher grades)
3+	Permits the observer to see the optic nerve head, but the borders are quite blurry
4+	Optic nerve head is obscured

[a] Grading should be conducted in a completely dark room.

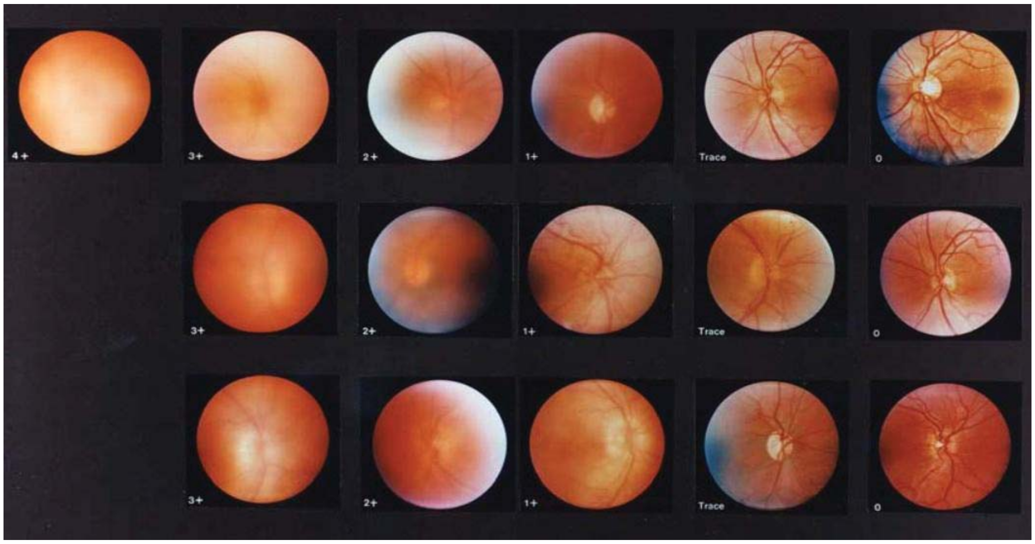

From Nussenblatt et al. Standardization of vitreal inflammatory activity in intermediate and posterior uveitis. Ophthalmology 1985;92:467-471.

A Pragmatic, Randomized Clinical Trial of Coronavirus (COVID-19) Vaccinations in Uveitis

Study Protocol

Version Number: 1.0

The First Affiliated Hospital of Chongqing Medical University,

Chongqing Key Laboratory of Ophthalmology and Chongqing Eye Institute,

Chongqing, China

Protocol Title: A Pragmatic, Randomized Clinical Trial of Coronavirus (COVID-19) Vaccinations in Uveitis

Version Number: 1.0

Protocol Date: July 1st, 2021

Site: Single center (The First Affiliated Hospital of Chongqing Medical University)

Grant: Chongqing Key Laboratory of Ophthalmology (CSTC, 2008CA5003) and National Natural Science Foundation of China

Chief Investigator: Peizeng Yang, MD., PhD

Chief Investigator's Correspondence: Department of Ophthalmology, The First Affiliated Hospital of Chongqing Medical University, Chongqing 400016, China; Tel: [redacted]; E-mail: peizengycmu@126.com

Back-up 24 Hour Global Emergency Contact Call: [redacted]

The back-up 24 hour global emergency contact call should only be used if you are not able to reach the Clinical Research Physician(s) or Study Coordinator(s) for emergency calls.

CONTENTS

CONTENTS 3

ABBREVIATIONS 6

1 Introduction 7

1.1 Background 7

1.2 Benefits and Risks 9

2 Objectives 11

2.1 Trial Objectives 11

2.2 Primary Objective 11

2.3 Secondary Objectives 11

2.4 Safety Objectives 11

3 Study Design 12

3.1 Overall Design 12

3.2 Study Schematic 12

3.3 Study Timeline 13

4 Eligibility Criteria 14

4.1 Inclusion Criteria 14

4.2 Exclusion Criteria 14

4.3 Re-screening of Patients 15

5 Study Procedures 16

5.1 Entering Screening 16

5.2 Eligibility Evaluation 16
5.3 Randomization Assignment and Masking 16
5.4 Baseline Assessments 17
5.5 Interventions 17
5.6 Follow-Up 18
5.7 Unscheduled Visits 24
5.8 Withdrawn and Lost to Follow-up 24
5.9 Special Ocular Examinations 25
5.10 Study Schedule Overview 27
6 Outcome Assessments 28
6.1 Adjudication Committee 28
6.2 Primary Outcome 28
6.3 Secondary Outcomes 28
6.4 Systemic Outcomes 30
6.5 Pre-specified Subgroup Analyses 30
7 Adverse Events 31
7.1 Adverse Event Reporting 31
7.2 Serious Adverse Events 31
7.3 Severity Assessments 32
7.4 Causality Assessments 32
7.5 Uveitis-Related Events 32

7.6 Pregnancy 33

7.7 Management 33

8 Data Collection and Management 35

8.1 Confidentiality 35

8.2 Source and Data Collection 35

8.3 Quality Assurance 35

8.4 Database Lock 36

8.5 Archiving 36

9 Statistical Considerations 37

9.1 Sample Size Calculation 37

9.2 Data Analyses 37

9.3 Interim Analysis 38

10 Ethical Considerations 39

REFERENCES 40

Appendix I 41

Appendix II 42

ABBREVIATIONS

AE	Adverse event
BCVA	Best corrected visual acuity
CFDA	China Food and Drug Administration
COVID-19	Coronavirus Disease 2019
CRF	Case report form
FFA	Fluorescein fundus angiography
NEI	National Eye Institute
OCT	Optical coherence tomography
SUN	Standardization of Uveitis Nomenclature Working Group

1 Introduction

1.1 Background

Why do we need a vaccine for COVID-19?

As the Coronavirus Disease 2019 (COVID-19) continues to affect and kill thousands of people around the world, politicians, health professionals and the public want to know how to get out of the blockade and begin to return to normal life. Without a vaccine against SARS-COV-2, the coronavirus that causes COVID-19, there is always a risk of new disease outbreaks. While rigorous testing, contact tracking, and isolation procedures will help control the spread of COVID-19, the only way to significantly reduce the threat is to immunize a large enough population against the virus (often known as herd immunity) so that the population cannot transmit it.[1,2] To achieve this, it is estimated that about 60% of the population to be immune to the disease[1,2] Everyone 18 years of age and older is now eligible to get a COVID-19 vaccination. Widespread vaccination is a critical tool to help stop the pandemic.

Do people with underlying medical conditions need a vaccine for COVID-19?

Adults of any age with certain underlying diseases are at increased risk of developing serious diseases due to the virus that causes COVID-19. The COVID-19 vaccine is recommended and can be administrated in most people with underlying diseases. People with autoimmune conditions may receive a COVID-19 vaccine. However, we should be aware that no data are currently available for this population. People from this group were eligible for enrollment in some of the clinical trials. However, evidence for the timing of COVID-19 vaccination in patients with autoimmune disease is still lacking.

What is uveitis?

Uveitis is one of several vision-threatening diseases. Originally, uveitis refers to a

collection of diseases characterized by intraocular inflammation that mainly affect the uvea, consisting of the iris, ciliary body and choroid. Uveitis also encompasses a range of entities involving inflammation of adjoining intraocular structures including the retina, vitreous and optic nerve.[3] According to the major site of inflammation, uveitis can be anatomically classified into anterior uveitis (inflammation of the iris and anterior chamber), intermediate uveitis (inflammation of the ciliary body and vitreous body), posterior uveitis (inflammation of the vitreous body, retina, choroid or even the optic disk) and panuveitis (a diffuse inflammation from the anterior through the posterior segment of the eye).[4]

Uveitis can also be classified into two categories, infectious or non-infectious, and the standard treatments for the two categories are largely different. Infectious uveitis can be caused by a localized or systemic infection with a definite pathogens, such as cytomegalovirus, herpes simplex virus (HSV), *Toxoplasma gondii, Treponema pallidum* and *Mycobacterium tuberculosis*, which requires anti-infection therapy.[5] Non-infectious uveitis can be associated with systemic diseases including spondyloarthritis, Behçet's disease, Vogt-Koyanagi-Harada disease (VKH) and sarcoidosis. In addition, non-infectious uveitis may be confined to the eye with no apparent associated systemic diseases, such as sympathetic ophthalmia, birdshot chorioretinopathy, serpiginous choroiditis, or multiple evanescent white dot syndrome.[6] Most non-infectious uveitis entities are presumed to be an immune-related disorder, usually termed as autoinflammatory or autoimmune disease, which means a self-directed pathological process. In clinical practice, these entities are primarily treated with immunosuppressive agents to suppress the autoinflammatory or autoimmune responses.[7]

What is the concern on vaccination in uveitis?

Having uveitis is currently not a contraindication (a medical reason to avoid) the vaccine. In fact, getting the vaccine when having uveitis may be especially important,

given the disease may confer a higher risk for COVID-19 or severe outcomes. However, it is possible that individuals could have an immune response to the COVID-19 vaccine. It remains unclear whether this immune response may significantly impact on the outcome of patients with uveitis. Furthermore, uveitis may also be a possible adverse side effect of some other vaccines.[8-10] Given the fact that patients with uveitis are on immunosuppressants like steroids, methotrexate, or biologics, they may be on different disease status (active, inactive or remission), and they have different causes (infectious or non-infectious), it is unknown about the best timing of COVID-19 vaccination which can cause minimal adverse events on uveitis patients.

What is the key question of our study?

In this study, we aim to initiate a pragmatic randomized trial to investigate whether there is significant difference in outcomes of patients with an inactive uveitis (active uveitis is considered not eligible for COVID-19 vaccination) between prompt initiation of COVID-19 vaccination and deferred initiation of COVID-19 vaccination until uveitis remission.

1.2 Benefits and Risks

The risks to participants are minimal with randomizing COVID-19 vaccination recommendations as part of clinical care, as it is simply testing two different vaccination strategies. The primary potential risk will be uveitis relapse. However, uveitis relapse is common and can be properly treated, and relapse related visual loss can be adequately and timely prevented provided a close monitoring. To minimize potential risk, we will implement best medical judgment and treatment if any study endpoint event occurs to assure the safety of participants.

The potential for public health benefit from our aims to randomize COVID-19 vaccination recommendations is of importance, potentially to the benefit of future

COVID-19 vaccination recommendation and decision in patients with uveitis. This study would provide direct evidence and timely data to guide physician to give proper recommendations on COVID-19 vaccination for patients with uveitis.

2 Objectives

2.1 Trial Objectives

The objective is to test the difference in ocular and systemic outcomes between prompt initiation of COVID-19 vaccination and deferred initiation of COVID-19 vaccination in patients with uveitis.

2.2 Primary Objective

To compare the time to symptomatic uveitis worsening between prompt and deferred vaccination strategies for patients with uveitis.

2.3 Secondary Objectives

To compare vaccine accessibility, patient compliance, clinically-confirmed uveitis worsening, visual outcomes, treatment interruptions and initiation of step-up treatment, hospitalization, laboratory-confirmed COVID-19 between prompt and deferred vaccination strategies for patients with uveitis.

2.4 Safety Objectives

To evaluate the systemic adverse events of the prompt versus deferred vaccination strategies for patients with uveitis.

3 Study Design

3.1 Overall Design

This is a pragmatic, randomized clinical trial to test the difference in ocular and systemic outcomes between prompt initiation of COVID-19 vaccination and deferred initiation of COVID-19 vaccination in uveitis patients who have not yet been vaccinated and whose ocular disease maintains inactive but has not yet achieved complete remission.

Approximately 1314 eligible subjects will be randomized 1:1 (657 subjects per group) to receive one of two vaccination strategy recommendations. In the prompt vaccination arm, subjects will be recommended to get a prompt initiation of COVID-19 vaccination. In the deferred vaccination arm, subjects will be recommended to get the vaccination after uveitis remission (inactive disease for 3 months after discontinuing all treatments for eye disease). All participants will be followed up for 3 months since randomization. The primary outcome will be the difference in time to symptomatic uveitis worsening.

3.2 Study Schematic

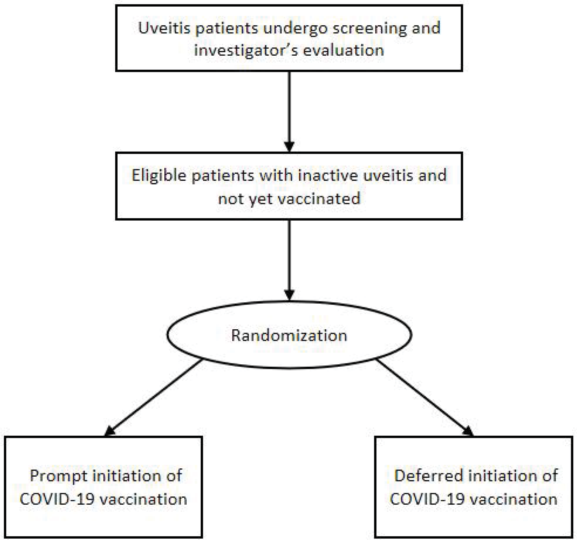

3.3 Study Timeline

Expected time for trial start: July 2021

Expected completion time of enrollment: October 2021

Expected time for end of trial: January 2022

The end of trial refers to the date of the last visit of the last subject to complete the study, or the date of receipt of the last data point from the last subject that is required for primary, secondary and/or exploratory analysis, as pre-specified in the protocol and/or the Statistical Analysis Plan, whichever is the later date.

Expected completion time of primary analysis: May 2022

4 Eligibility Criteria

An important goal of pragmatic randomized trials is to provide relevant and generalizable information to guide clinical decision-making. As such, the inclusion and exclusion criteria of pragmatic trials is often more broad than those of traditional randomized controlled trials.

4.1 Inclusion Criteria

A subject will be eligible for study participation if she/he meets all of the following inclusion criteria:

a. Aged 18 years or older

b. Not yet vaccinated prior to the Screening.

c. Diagnosed with any forms of uveitis in any eye.

d. An **inactive disease** status (all the following criteria are met):

   (1) Anterior chamber cells of 0.5+ or less (See SUN criteria[11] in Appendix I);
   (2) Vitreous haze of 0.5+ or less (See NEI/SUN criteria[12] in Appendix II);
   (3) No active inflammatory choroidal or retinal vascular lesions.

4.2 Exclusion Criteria

The presence of any of the following will exclude a subject from the study enrollment:

a. Complete uveitis remission: Inactive disease for 3 months after discontinuing all treatments for eye disease.

b. Any known vaccine contraindications according to manufactures' instructions:

   (1) Individuals with a history of anaphylaxis to any component of the vaccine;
   (2) Individuals with a body temperature over 38.5°C;
   (3) Pregnancy.

c. Any other condition which, in the opinion of the Investigator, would put the subject at risk by participation in the protocol.

4.3 Re-screening of Patients

Individuals that do not meet the above eligibility criteria may be re-screened a minimum of 2 weeks after the last screening visit.

5 Study Procedures

5.1 Entering Screening

Procedures for obtaining consent include explaining the patient's disease, prognosis, and options, introducing the aims and methods of this trial, discussing the risks and benefits of participation and addressing the patient's questions and concerns. Patients will be given at least 24 hours to consider taking part. The subject is assured that participation in this study is voluntary and he/she can withdraw at any time, without giving a reason. A signed informed consent will be obtained from the subject or their legally authorized representative before any study-related procedures are undertaken.

5.2 Eligibility Evaluation

The eligibility evaluation will be performed at the Screening. For detailed inclusion/exclusion criteria see Section 4.

The Investigator will perform slit-lamp biomicroscopy, ophthalmoscopy and visual acuity on each candidate during the Screening. Other auxiliary examinations including fundus photography, optical coherence tomography (OCT) and fundus fluorescence angiography (FFA), may be performed at the Investigators' discretion according to the clinical needs to evaluate patient's ocular condition, and examination results or reports will be recorded on medical notes.

5.3 Randomization Assignment and Masking

After eligibility has been determined, assignment of a randomization takes place at the Baseline visit. Only subjects who meet all of the inclusion criteria and none of the exclusion criteria are eligible for randomization into the treatment phase. Randomization will be designed to yield expected assignment ratio of 1:1 to the prompt vaccination strategy and the deferred vaccination strategy. Randomization list will have been generated by a Statistician using a computer and will be properly kept

by a Designated Study Coordinator. Both this Statistician and the Designated Study Coordinator will have no involvement in other parts of the trial. The Investigator who is designated as the Primary Treating Ophthalmologist and is responsible for the management of the subject in this trial will telephone the Designated Study Coordinator to obtain patient assignment information. Throughout the trial, the randomization assignment will not be masked to the Primary Treating Ophthalmologist, but the Primary Treating Ophthalmologist will not have direct access to the randomization list. The randomization assignment will be masked to an adjudication committee who will independently determine the study endpoint events according to medical data, records and examination reports written by the Primary Treating Ophthalmologist, the attending doctor or the examining doctor. **These medical notes and data will be collected by a group of Study Coordinators who mask any randomization assignment information and submit these materials online to the adjudication committee.**

5.4 Baseline Assessments

The Baseline visit becomes time-point zero from which all other visit dates are calculated. The Screening and the Baseline can be performed on the same day. Considering the pragmatic study design, to ensure a rapid enrollment and data collection for a broad range of participants, baseline clinical characteristics and medical history can be either reported by patients or retrieved by means of a study site-specific query of the health record. Pre-specific baseline data include the date of birth, gender, ethnic group, height, weight, presence of hypertension, diabetes, coronary heart disease, malignancies, type of uveitis (anterior, intermediate, posterior or panuveitis), specific uveitis diagnosis, affected eye (right, left or both), duration of uveitis, number of flares in the past 12 month, best corrected visual acuity logMAR score, and intraocular pressure.

5.5 Interventions

Interventions will take place at the Baseline visit. The Primary Treating Ophthalmologist will provide the recommendation of COVID-19 vaccination to the subject according to randomization assignment. For those randomized to the prompt vaccination, subjects will be recommended to go to vaccination site and get COVID-19 vaccination as soon as possible. Our ophthalmologic clinic does not provide the vaccination service. We will only give the recommendation to patients. For those randomized to the deferred vaccination, subjects will be recommended to get COVID-19 vaccination after the remission of uveitis. **Remission is defined as an inactive disease for 3 months after discontinuing all treatments for eye disease.** At baseline, all patients will also be educated to immediately contact us by phone call once they experience any discomfort or have any new ocular symptoms of eye redness, eye pain, decreased vision, light sensitivity or fark, floating spots. We will ask questions as prespecified in **5.6 Follow-up** on the phone call.

5.6 Follow-Up

Routine follow-up encounters will then occur every month. Participants will complete trial visits primarily by telephone calls from the call center. However, all participants will need to complete an in-person clinic visit on the end of three months since baseline.

On phone call, patients will be asked to answer the following predetermined questions according to their randomization assignment. **Module 1** will be subjected to those in prompt vaccination group, and **Module 2** will be subjected to those in deferred vaccination group.

Module 1

Question 1: Do you think you have had a uveitis worsening or disease recurrence (relapse) in the past month?

Yes, no or uncertain. If yes, specify which eye and duration.

Question 2: Have you ever experienced newly occurred eye redness in the past month?

Yes, no or uncertain. If yes, specify which eye and duration.

Question 3: Have you ever experienced newly occurred eye pain in the past month?

Yes, no or uncertain. If yes, specify which eye and duration.

Question 4: Have you ever experienced new blurred vision or decreased vision in the past month?

Yes, no or uncertain. If yes, specify which eye and duration.

Question 5: Have you ever experienced newly occurred light sensitivity in the past month?

Yes, no or uncertain. If yes, specify which eye and duration.

Question 6: Have you ever had newly occurred dark, floating spots in your field of vision (floaters, specks or moving clouds in your vision) in the past month?

Yes, no or uncertain. If yes, specify which eye and duration.

Question 7: Have you ever experienced other ocular or systemic symptoms in the past month?

Yes, no or uncertain. If yes, specify where and duration.

Question 8: Have you already been received any dose of COVID-19 vaccination?

Yes or no. If no, jump to Question 14.

Question 9: Which COVID-19 vaccine did you get?

Sinovac (CoronaVac), Sinopharm, ZFSW Biologics Company, others (specifically), or uncertain.

Question 10: How many doses of COVID-19 vaccines have you already been

received?

One, two, three or more (specific numbers).

Question 11: When did you get each dose of COVID-19 vaccines?

Participants will be asked to provide the specific date as far as possible.

Question 12: Have you finished all required doses of COVID-19 vaccines?

Yes or no. If the answer is no, continue on Question 13, otherwise finished.

Question 13: When do you plan to get the next vaccine?

Participants will be asked to provide the specific date as far as possible. If participants answer they no longer intend to get the next vaccine, specific reasons should be provided. Questions finished.

Question 14: Why did not you get vaccinated?

Patients will be asked to provide the reason for not having COVID-19 vaccination. Not willing to get vaccination, willing to get vaccination but having not yet gone to the vaccination site (specific reasons for not yet gone to the vaccination site required), having gone to the vaccination site but refused due to the lack of vaccines, having gone to the vaccination site but refused due to contraindication, other reasons (specifically). After completing this question, all participants will be encouraged to go to get vaccination as soon as possible on the phone, although they might have shown their unwillingness to get one or they might have tried but failed.

At the end of phone call, if the patient answer yes to any of questions 1 to 7 in the Module 1, the patient will be instructed to come back to study site to complete in-person clinic visit for further evaluation and examination. Alternatively, the patient will also be allowed to go to local hospitals for evaluation and examination, but the patient need to send back the copies of all medical notes, records, laboratory or auxiliary examination reports from the local hospitals. **Participants will receive best**

medical judgment and proper treatment for worsening of the ocular or systemic conditions, including but not limited to step-up treatment, treatment interruptions even hospitalization. Nasopharyngeal swabs for RT-PCR testing of SARS-COV-2 will be performed on cases of hospitalization. No matter where the evaluation is completed, patients will be instructed to undergo three mandatory ocular assessments, including **best-corrected visual acuity testing, slit-lamp biomicroscopy and ophthalmoscopy**. Mandatory materials send back from local hospitals include the medical record written by the attending doctor with signature and the prescription of that clinic visit. An adjudication committee who are unaware of group assignment will independently determine the endpoint according to these medical records from our study site or the local hospitals. **If clinically-confirmed uveitis worsening occurs, participants will be recommended to postpone subsequent vaccination until uveitis remission.**

Module 2

Question 1: Do you think you have had a uveitis worsening or disease recurrence (relapse) in the past month?

Yes, no or uncertain. If yes, specify which eye and duration.

Question 2: Have you ever experienced newly occurred eye redness in the past month?

Yes, no or uncertain. If yes, specify which eye and duration.

Question 3: Have you ever experienced newly occurred eye pain in the past month?

Yes, no or uncertain. If yes, specify which eye and duration.

Question 4: Have you ever experienced new blurred vision or decreased vision in the past month?

Yes, no or uncertain. If yes, specify which eye and duration.

Question 5: Have you ever experienced newly occurred light sensitivity in the past month?

Yes, no or uncertain. If yes, specify which eye and duration.

Question 6: Have you ever had newly occurred dark, floating spots in your field of vision (floaters, specks or moving clouds in your vision) in the past month?

Yes, no or uncertain. If yes, specify which eye and duration.

Question 7: Have you ever experienced other ocular or systemic symptoms in the past month?

Yes, no or uncertain. If yes, specify where and duration.

Question 8: Have you been disease-free and discontinuing uveitis treatment for 3 months?

Yes or no. If yes, jump to Question 15.

Question 9: Have you already been received any dose of COVID-19 vaccination?

Yes or no. If no, participants will be recommended to keep away from getting vaccination at present and the conversation will be finished, otherwise continue on Question 10.

Question 10: Which COVID-19 vaccine did you get?

Sinovac (CoronaVac), Sinopharm, ZFSW Biologics Company, others (specifically), or uncertain.

Question 11: How many doses of COVID-19 vaccines have you already been received?

One, two, three or more (specific numbers).

Question 12: When did you get each dose of COVID-19 vaccines?

Participants will be asked to provide the specific date as far as possible.

Question 13: Have you finished all required doses of COVID-19 vaccines?

Yes or no. If the answer is no, continue on Question 14, otherwise finished.

Question 14: When do you plan to get the next vaccine?

Participants will be asked to provide their plan on specific date as far as possible. If participants answer they no longer intend to get the next vaccine, specific reasons should be provided. Questions finished.

Question 15: Have you already been received any dose of COVID-19 vaccination?

Yes or no. If yes, back to Question 10. If no, jump to Question 16.

Question 16: Why did not you get vaccinated?

Patients will be asked to provide the reason for not having COVID-19 vaccination. Not willing to get vaccination, willing to get vaccination but having not yet gone to the vaccination site (specific reasons for not going to the vaccination site required), having gone to the vaccination site but refused due to the lack of vaccines, having gone to the vaccination site but refused due to contraindication, other reasons (specifically). After completing this question, all participants will be encouraged to go to get vaccination as soon as possible on the phone, although they might have shown their unwillingness to get one.

At the end of phone call, if the patient answer yes to any of questions 1 to 7 in the Module 2, the patient will be instructed to come back to study site to complete in-person clinic visit for further evaluation and examination. Alternatively, the patient will also be allowed to go to local hospitals for evaluation and examination, but the patient need to send back the copies of all medical notes, records, laboratory or auxiliary examination reports from the local hospitals. **Participants will receive best medical judgment and proper treatment for worsening of the ocular or systemic**

conditions, including but not limited to step-up treatment, treatment interruptions even hospitalization. Nasopharyngeal swabs for RT-PCR testing of SARS-COV-2 will be performed on cases of hospitalization. No matter where the evaluation is completed, patients will be instructed to undergo three mandatory ocular assessments, including **best-corrected visual acuity testing, slit-lamp biomicroscopy and ophthalmoscopy**. Mandatory materials send back from local hospitals include the medical record written by the attending doctor with signature and the prescription of that clinic visit. An adjudication committee who are unaware of group assignment will independently determine the endpoint according to these medical records from our study site or the local hospitals. **If clinically-confirmed uveitis worsening occurs, participants will be recommended to postpone subsequent vaccination until uveitis remission. If a patient assigned to the deferred vaccination group is vaccinated without remission of his/her ocular disease, we will no longer provide any recommendations on vaccination and implement assigned intervention strategies, this patient will only be observed later.**

5.7 Unscheduled Visits

Participants will encounter at the clinic and have unscheduled visit for any reasons. They will be asked to answer questions in Module 1 or Module 2 according to their assignment. They will also undergo three mandatory assessments, including **best-corrected visual acuity testing, slit-lamp biomicroscopy and ophthalmoscopy,** and other examinations at investigator's discretion. For those with disease worsening, participants will receive best medical judgment and proper treatment for worsening of the ocular or systemic conditions.

5.8 Withdrawn and Lost to Follow-up

Subjects have the right to withdraw from the trial at any time and for any reason,

without providing a reason. The investigator has the right to withdraw participants from the study and abandon assigned intervention strategies in the event of inter-current illness, adverse events, protocol violations or other reasons.

If the subject withdraws from the trial, efforts will be made to continue to obtain follow-up data, with the permission of the subject. They will be given appropriate treatment, but will not continue with scheduled study follow-ups unless they allow it.

Subjects will also be considered to have been withdrawn if they declare they are no longer interested in further participation, or have died. If the death is considered to be related to the vaccines, the subject will be declared serious adverse event, rather than withdrawn.

Missing a phone call or visit does not necessarily mean that a patient has been withdrawn from the study. For these subjects, efforts will be made to contact them and bring them back as soon as possible.

For those subjects fail to response the contact or to attend study visits without stating an intention to withdraw from the study, the Investigator should contact the subject through telephone calls as soon as possible. The investigator will attempt to contact them for at least three times at each scheduled visit. If contact cannot be established up to the end of the trial, the subject should be considered as “lost to follow-up” on the date of last contact.

5.9 Special Ocular Examinations

Visual acuity testing

With the appropriate corrective lenses based on that subject’s refraction, the best corrected visual acuity (BCVA) will be measured using a Logarithmic visual acuity chart or according to local routine practice. The subject’s presenting visual acuity

without refraction will also be evaluated. Visual acuity will be obtained prior to pupil dilation. Data will be transformed into LogMAR score.

Tonometry

Intraocular pressure for both eyes will be measured using a non-contact tonometry. Tonometry should be performed prior to pupil dilation.

Slit-lamp biomicroscopy

A standard ophthalmic examination using slit lamp biomicroscopy will be performed in both eyes by the unmasked Primary Treating Ophthalmologist. The Anterior chamber cell count will be measured during the examination according to the SUN criteria (see Appendix I), which should be performed prior to pupil dilation.

Ophthalmoscopy

Ophthalmoscopy will be performed by the Primary Treating Ophthalmologist under the dilation of the pupil to determine both vitreous haze grading and the absence/presence of active chorioretinal and/or retinal vascular lesions. Grading of vitreous haze will be based on the publication from the National Eye Institute (NEI) which has also been adapted by the SUN working group (see Appendix II).

Fundus photography

Fundus photography will be performed to obtain evidence of the absence/presence of active chorioretinal and/or retinal vascular lesions.

Optical coherence tomography (OCT)

Optical coherence tomography will be used to determine the absence/presence of retinal lesions and macular alternations.

Fundus fluorescence angiography (FFA)

Fundus fluorescence angiography will be performed to determine the

absence/presence of active chorioretinal and/or retinal vascular lesions.

5.10 Study Schedule Overview

	**Screening/Baseline**	**Month 1 (phone call)**	**Month 2 (phone call)**	**Month 3 (in person)**	**End trial month after Month 3 (phone call)**	**Post-symptom visit (our study site or local hospital)**	**Unscheduled visit (in person)**
Visit window (day)	0	±7	±7	±14	±7	-	-
Written informed consent	×						
Inclusion/exclusion criteria	×						
Randomization	×						
General information and medical history	×						
Answer to Questions (Module 1 or Module 2)		×	×	×*	×		×
Visual acuity testing	×			×		×	×
Slit-lamp biomicroscopy	×			×		×	×
Ophthalmoscopy	×			×		×	×
Any other necessary auxiliary examination at discretion	×			×		×	×
Treatment, prescription and record	×			×		×	×
Adverse event evaluation		×	×	×	×	×	×

* can be assessed by phone call.

6 Outcome Assessments

6.1 Adjudication Committee

An adjudication committee who are unaware of group assignment will independently determine each endpoint event.

6.2 Primary Outcome

The primary outcome will be the time to symptomatic worsening over the course of 3 months of follow-up.

Symptomatic worsening is defined if one of following newly onset symptoms occurs in at least one eye lasting for at least 2 days: eye redness, eye pain, decreased vision, light sensitivity or floaters.

6.3 Secondary Outcomes

a. Time to clinically-confirmed uveitis worsening.

Clinically-confirmed uveitis worsening is defined if any of the following criteria are met in at least one eye: new active, inflammatory chorioretinal or retinal vascular lesions (determined by clinical examination such as ophthalmoscopy or ancillary testing such as fundus photography and fluorescein angiography); worsening of best corrected visual acuity by 0.3 LogMAR score or more; a two-step increase in anterior chamber cell grade relative to baseline; or a two-step increase in vitreous haze grade relative to baseline. *A 2-step increase is represented by a change of grade 0 to grade 2+; or grade 0.5+ to grade 3+.*

b. Proportion of each reason for symptomatic uveitis worsening.

c. Proportion of symptomatic uveitis worsening.

d. Proportion of each reason for clinically-confirmed uveitis worsening.

e. Time to initiation of step-up treatment.

Step-up treatment is defined if any of the following criteria are met: addition of another systemic immunosuppressive agent (or biologics), switching to another systemic immunosuppressive agent (or biologics), or increasing doses of any currently systemically administrated drugs.

f. Time to treatment interruptions.

Treatment interruptions are defined if any of the following criteria are met: decreasing doses of or discontinuing any currently systemically administrated drugs due to adverse events rather than disease improvement.

g. Time to hospitalization for any reasons.

h. Time to hospitalization for ocular disease.

i. Time to laboratory-confirmed COVID-19.

j. Patient adherence, defined as proportion correctly vaccinated for at least one dose or maintaining not vaccinated per protocol throughout the trial.

k. Proportion of vaccinated type and dose.

l. Proportion of uveitis remission throughout the trial.

Uveitis remission is defined as an inactive disease for 3 months after discontinuing all treatments for eye disease.

m. Vaccine inaccessibility, defined as proportion having gone to the vaccination site but refused due to the lack of vaccines, whereby this situation continues until the end of trial.

n. Reasons for not yet vaccinated until the end of trial among those who should have been vaccinated per protocol.

o. Change in anterior chamber cells from the Baseline to the end of three months.

p. Change in vitreous haze from the Baseline to the end of three months.

q. Change in best corrected visual acuity LogMAR score from the Baseline to the end of three months.

6.4 Systemic Outcomes

The systemic outcome will be systemic adverse events (apart from uveitis related events).

6.5 Pre-specified Subgroup Analyses

To determine differences between assignment groups in outcomes across subgroup variable defined by uveitis anatomical subtypes, etiology diagnosis classification, history of uveitis flare, age and sex.

7 Adverse Events

7.1 Adverse Event Reporting

An adverse event (AE) is defined as any unfavorable medical occurrence in a subject who has ever received study intervention, regardless of a causal relationship with this intervention. Any worsening of a pre-existing condition or illness should be considered an adverse event. Worsening in severity of a reported adverse event should also be reported as a new adverse event. An elective surgery/procedure scheduled to occur during a study will not be considered an adverse event if the surgery/procedure is being performed for a pre-existing condition and the surgery/procedure has been pre-planned prior to study entry. However, if the pre-existing condition deteriorates unexpectedly during the study (e.g., surgery performed earlier than planned), then, the deterioration of the condition for which the elective surgery/procedure is being done will be considered an adverse event. For adverse events to be considered intermittent, the events must be of similar nature and severity.

The Investigator will monitor each subject for clinical and laboratory evidence of adverse events throughout the study. The Investigator will assess and record any adverse event in detail including the date of onset, event diagnosis (if known) or sign/symptom, severity, time course, duration and outcome, relationship of the adverse event to study intervention and any actions taken. All these information on non-serious and serious adverse events will be recorded at each study visit.

7.2 Serious Adverse Events

Serious adverse events (SAE) should be reported to Chief Investigator and Ethics Committee within 24 hours of the Investigator being made aware of the SAE. Serious adverse events include any medical occurrence that results in the following outcomes:

Death.

Life-threatening experience.

Non-elective surgery or hospitalization for any reason.

Congenital anomaly or birth defect.

Persistent or significant disability or incapacity.

Any other important medical events, not immediately life-threatening or resulting in death or hospitalization but requiring medical or surgical intervention to prevent serious outcome.

7.3 Severity Assessments

The severity of adverse event can be assessed according to the following criteria:

Mild: The adverse event does not interfere with the volunteer's daily routine, and does not require intervention; it causes slight discomfort.

Moderate: The adverse event interferes with some aspects of the volunteer's routine, or requires intervention, but is not damaging to health; it causes moderate discomfort.

Severe: The adverse event results in alteration, discomfort or disability which is clearly damaging to health.

7.4 Causality Assessments

The Investigator should assess the relationship of the adverse event with the use of vaccines by reporting according to the three-level classification of "associated", "not associated", and "inability to determine". Causality assessments will be required for study drugs as well as other adjunctive treatments.

7.5 Uveitis-Related Events

The following events are known complications related to the condition being treated and will be classified as uveitis-related events. These events will be analyzed

separately from other adverse events in the final study report. Notably, the following events may not be a complete list of all potential uveitis-related events. The Investigators must determine if a specific event is uveitis-related.

Loss of transparency of the cornea.

Band keratopathy.

Synechiae.

Cataracts.

Glaucoma/increased intraocular pressure.

Vitreous hemorrhage.

Macular edema.

Retinal detachment.

Epiretinal membrane.

Vitreo-macular traction.

Retinal ischemia.

Vision loss.

Hypotony.

7.6 Pregnancy

Subjects who become pregnant during the study period must be discontinued. Pregnancy in a study subject is not considered as an adverse event. However, the medical outcome of an elective or spontaneous abortion, stillbirth or congenital anomaly is considered as a serious adverse event.

7.7 Management

For the purpose of medical management, all adverse events and laboratory abnormalities that occur during the study must be evaluated by the Investigator and treated according to the best medical judgment of their clinician. The occurrence of adverse events does not necessarily mean the time for the subject to exit the study. But, a serious adverse event will absolutely result in the discontinuation of the study immediately. For those developing non-serious adverse events, administration of other adjunctive treatments or even stopping the assigned intervention may be used.

8 Data Collection and Management

8.1 Confidentiality

Participants will be identified via a unique ID. Identifiable information will not be stored in the eCRF and will not leave the site. Any participant contact information will be stored within the site on password protected computers or within secured locations with limited access. All study data and site files will be kept at site in a secure location with restricted access.

8.2 Source and Data Collection

The study will employ an eCRF, whereby data will be managed via this system. Source data worksheets will be used for each patient and data will be entered onto the eCRF database. Source data worksheets will be reconciled at the end of the trial with the patients' medical notes in the study site. During the trial, critical clinical information will be written in the medical notes to ensure informed medical decisions can be made in the absence of the study team. Trial related clinical letters will be copied to the medical notes during the trial. The Chief Investigator will provide a signature for CRF once all queries are resolved and immediately prior to database lock.

It will be the responsibility of the Chief Investigator and his team to ensure the accuracy of all data entered in the worksheets and the eCRF are in accordance with Good Clinical Practice. The Principal Investigator will be responsible for ensuring that source data worksheets are filed in a suitably secure location to ensure source data verification can be undertaken throughout the study.

8.3 Quality Assurance

The study will incorporate a range of data management quality assurance functions as the trial progresses. The Trial Data Manager will provide study training, ongoing

study support and will conduct regular monitoring, checking source data for transcription errors. Any necessary alterations to entered data will be date and time stamped within the eCRF. Regular monitoring of study conduct and data collected will be performed to ensure the study is conducted in accordance with GCP.

8.4 Database Lock

Prior to database lock, the Trial Manager will review any outstanding warnings on the eCRF and resolve or close these as appropriate before database lock. Local study personnel should resolve any queries that arise promptly. Once all queries have been resolved no further changes will be made to the database unless specifically requested by the Study Office in response to the Statistician's data checks. The study Chief Investigator will review all the data and provide electronic signature to verify that all the data are complete and correct. At this point, all data will be formally locked for analysis.

8.5 Archiving

Chief Investigator will be responsible for securely archiving local data generated, essential documents and source data in accordance with local requirements, but for at least 5 years from the end of the study.

9 Statistical Considerations

9.1 Sample Size Calculation

A two-sided log-rank test with an overall sample size of 1314 subjects (657 in the deferred vaccination group and 657 in the prompt vaccination group) achieves 80.0% power at a 0.050 significance level to detect a hazard ratio of 1.5 when the proportion surviving (no occurrence of symptomatic uveitis worsening) in the deferred vaccination group is 0.9000. The study lasts for 6 time periods of which subject accrual (entry) occurs in the first 3 time periods. The accrual pattern across time periods is uniform (all periods equal). The proportion dropping out of both groups is 0.1500, respectively.

9.2 Data Analyses

Analysis methods of each study outcome will be provided in a detailed statistical analysis plan.

Primary analyses will be on an Intention-to-treat (ITT) basis. According to the intention-to-treat principle (ITT), all patients who have been randomized regardless of their compliance with the study intervention and whose primary outcome is available will be included, which is one of the main data sets for the effectiveness evaluation of this study. Given the pragmatic nature of the trial and the expected gaps in data on patient compliance with randomized intervention, we will not consider the per-protocol (PP) analysis as the primary analysis.

The clinical and demographic characteristics of the participants will be summarized with descriptive statistics. Continuous variables will be described with their distribution range, mean, median, standard deviation, and interquartile range, as appropriate. Categorical variables will be summarized as numbers and the proportions.

The primary outcome will be the time to symptomatic uveitis worsening. For the primary outcome, the multiplicative limit method (Kaplan-Meier) is used for univariate survival analysis, the median time to symptomatic uveitis worsening and survival rate of each group are calculated, and the survival rate curve is compared by the Log-rank test.

For the systemic outcomes, the types, number, and incidence of all systemic adverse events during the follow-up period will be described in a tabular form.

9.3 Interim Analysis

This study does not plan to perform interim analysis.

10 Ethical Considerations

This clinical trial will be conducted in compliance with the approved protocol, the Helsinki Declaration, the Drug Clinical trial Management Code issued by the CFDA, and corresponding regulations.

Before the commencement of this experiment, the approval of the ethics committee must be obtained.

The Investigator is expected to take any immediate action required for the safety of any patient included in this study, even if this action represents a deviation from the protocol.

During the clinical study, any changes made to this trial protocol should be reported to the Ethics Committee and placed on record.

Subjects must provide informed consent to participate in the trial.

If the subject and his or her legal representative are illiterate, the informed consent process shall be attended by a witness, who shall sign the informed consent form after oral consent by the subject or his or her legitimate representative.

A copy of the informed consent form and the contact information for the researcher and the ethics committee must be provided to the patient.

The study site must be equipped with the necessary medical rescue equipment and first aid drugs for emergency issues.

REFERENCES

1. Fontanet A, Cauchemez S. COVID-19 herd immunity: where are we? Nat Rev Immunol 2020;20:583-4.

2. Randolph HE, Barreiro LB. Herd Immunity: Understanding COVID-19. Immunity 2020;52:737-41.

3. Krishna U, Ajanaku D, Denniston AK, Gkika T. Uveitis: a sight-threatening disease which can impact all systems. Postgrad Med J 2017;93:766-73.

4. Tsirouki T, Dastiridou A, Symeonidis C, et al. A focus on the epidemiology of uveitis. Ocul Immunol Inflamm 2018;26:2-16.

5. Deschenes J, Murray PI, Rao NA, Nussenblatt RB. International Uveitis Study Group (IUSG): clinical classification of uveitis. Ocul Immunol Inflamm 2008;16:1-2.

6. Hsu Y-R, Huang JC-C, Tao Y, et al. Noninfectious uveitis in the Asia–Pacific region. Eye 2019;33:66-77.

7. Jabs DA, Rosenbaum JT, Foster CS, et al. Guidelines for the use of immunosuppressive drugs in patients with ocular inflammatory disorders: recommendations of an expert panel. Am J Ophthalmol 2000;130:492-513.

8. Esmaeli-Gutstein B, Winkelman JZ. Uveitis associated with varicella virus vaccine. Am J Ophthalmol 1999;127:733-4.

9. Heydari-Kamjani M, Vante I, Uppal P, Demory Beckler M, Kesselman MM. Uveitis Sarcoidosis Presumably Initiated After Administration of Shingrix Vaccine: Cureus. 2019 Jun 17;11(6):e4920. doi: 10.7759/cureus.4920.; 2019.

10. Cunningham ET, Jr., Moorthy RS, Fraunfelder FW, Zierhut M. Vaccine-Associated Uveitis: Ocul Immunol Inflamm. 2019;27(4):517-520. doi: 10.1080/09273948.2019.1626188.; 2019.

11. Jabs D, Nussenblatt R, Rosenbaum J, Standardization of Uveitis Nomenclature (SUN) Working Group. Standardization of uveitis nomenclature for reporting clinical data. Results of the first international workshop. Am J Ophthalmol 2005;140:509-16.

12. Nussenblatt RB, Palestine AG, Chan CC, Roberge F. Standardization of vitreal inflammatory activity in intermediate and posterior uveitis. Ophthalmology 1985;92:467-71.

Appendix I

SUN Working Group Grading System for Anterior Chamber Cells

Grade	Number of cells in field [a]
0	<1
0.5+	1-5
1+	6-15
2+	16-25
3+	26-50
4+	>50

[a] Field size is a 1mm by 1mm slit lamp beam. Grading should be done with the highest magnification and illumination and conducted in a completely dark room, prior to dilation.

From Jabs DA et al. Standardization of uveitis nomenclature for reporting clinical data. Results of the First International Workshop. Am J Ophthalmol 2005;140(3):509-16.

Appendix II

National Eye Institute (NEI)/SUN Criteria for Grading Vitreous Haze

Grade	Description [a]
0	No evident vitreal haze
0.5+	Slight blurring of the optic disc margin because of the haze; normal striations and reflex of the nerve fiber layer cannot be visualized
1+	Permits a better definition of both the optic nerve head and the retinal vessels (compared to higher grades)
2+	Permits better visualization of the retinal vessels (compared to higher grades)
3+	Permits the observer to see the optic nerve head, but the borders are quite blurry
4+	Optic nerve head is obscured

[a] Grading should be conducted in a completely dark room.

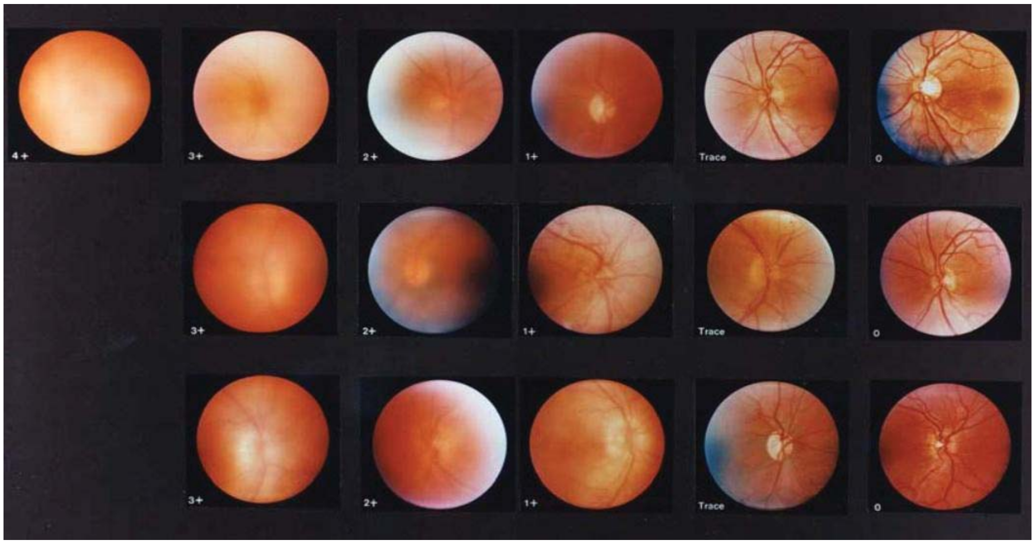

From Nussenblatt et al. Standardization of vitreal inflammatory activity in intermediate and posterior uveitis. Ophthalmology 1985;92:467-471.

Statistical Analysis Plan

A Pragmatic, Randomized Clinical Trial of Coronavirus (COVID-19) Vaccinations in Uveitis

Version 1.0   July 1st, 2021

The First Affiliated Hospital of Chongqing Medical University

Chongqing Key Laboratory of Ophthalmology and Chongqing Eye Institute

Chongqing, China

CONTENTS

1 Introduction 2
2 Study Objective 2
2.1 Primary Objective 2
2.2 Secondary Objectives 3
2.3 Safety Objectives 3
3 Study Outcomes 3
3.1 Primary Outcome 3
3.2 Secondary and Safety Outcomes 3
4 Statistical Hypothesis 5
5 Sample Size 6
6 Randomization and Masking 6
7 Analysis Population 6
7.1 Intention-To-Treat Population 6
7.2 Per Protocol Population 7
8 Missing Data 7
9 Baseline Characteristic Analysis 7
10 Primary Outcome Comparison 8
11 Analysis of Secondary Outcomes 8
11.1 General Principles 8
11.2 Analytical Methods 9
12 Subgroup Analysis 12
13 Sensitivity Analysis Evaluating Non-Response 12
14 Interim Analysis 13
15 Statistics Software 13
16 References 13

1 Introduction

This is an investigator-initiated, parallel group-randomized, pragmatic trial to test the difference in ocular and systemic outcomes between prompt initiation of COVID-19 vaccination and deferred initiation of COVID-19 vaccination in patients with uveitis.

Approximately 1314 eligible subjects will be randomized 1:1 (657 subjects per group) to receive one of two vaccination strategy recommendations. In the prompt vaccination arm, subjects will be recommended to get a prompt initiation of COVID-19 vaccination. In the deferred vaccination arm, subjects will be recommended to get the vaccination after uveitis remission (inactive disease for 3 months after discontinuing all treatments for eye disease). All participants will be followed up for 3 months since randomization. The primary outcome will be the difference in time to symptomatic uveitis worsening.

The statistical analysis plan (SAP) documents the planned statistical analyses for the trial and is based on the protocol, together with any subsequent amendments. The SAP is intended for the use of project team members and should be read in conjunction with the aforementioned protocol. The analyses that are detailed in this SAP will be performed only when the database has been locked and all protocol violators identified. Analysis populations will be determined using the rules prespecified in this SAP. At a date to be agreed within the project team, a data look and further analysis will be performed.

2 Study Objective

2.1 Primary Objective

To compare the time to symptomatic uveitis worsening between prompt and deferred vaccination strategies for patients with uveitis.

2.2 Secondary Objectives

To compare vaccine accessibility, patient compliance, clinically-confirmed uveitis worsening, visual outcomes, treatment interruptions and initiation of step-up treatment, hospitalization, laboratory-confirmed COVID-19 between prompt and deferred vaccination strategies for patients with uveitis.

2.3 Safety Objectives

To evaluate the systemic adverse events of the prompt versus deferred vaccination strategies for patients with uveitis.

3 Study Outcomes

3.1 Primary Outcome

An adjudication committee who are unaware of group assignment will independently determine each endpoint event according to telephone call records from the call center, medical data, records and examination reports written by the follow-up staff, the primary treating ophthalmologist, the attending doctor or the examining doctor. These medical notes and data will be collected by a group of Study Coordinators who mask any randomization assignment information and submit these materials to the adjudication committee.

The primary outcome will be the time to symptomatic worsening over the course of 3 months of follow-up.

Symptomatic worsening is defined if one of following newly onset symptoms occurs in at least one eye lasting for at least 2 days: eye redness, eye pain, decreased vision, light sensitivity or floaters.

3.2 Secondary and Safety Outcomes

a. Time to clinically-confirmed uveitis worsening.

Clinically-confirmed uveitis worsening is defined if any of the following criteria are met in at least one eye: new active, inflammatory chorioretinal or retinal vascular lesions (determined by clinical examination such as ophthalmoscopy or ancillary testing such as fundus photography and fluorescein angiography); worsening of best corrected visual acuity by 0.3 LogMAR score or more; a two-step increase in anterior chamber cell grade relative to baseline; or a two-step increase in vitreous haze grade relative to baseline. *A 2-step increase is represented by a change of grade 0 to grade 2+; or grade 0.5+ to grade 3+.*

b. Proportion of each reason for symptomatic uveitis worsening.

c. Proportion of symptomatic uveitis worsening.

d. Proportion of each reason for clinically-confirmed uveitis worsening.

e. Time to initiation of step-up treatment.

Step-up treatment is defined if any of the following criteria are met: addition of another systemic immunosuppressive agent (or biologics), switching to another systemic immunosuppressive agent (or biologics), or increasing doses of any currently systemically administrated drugs.

f. Time to treatment interruptions.

Treatment interruptions are defined if any of the following criteria are met: decreasing doses of or discontinuing any currently systemically administrated drugs due to adverse events rather than disease improvement.

g. Time to hospitalization for any reasons.

h. Time to hospitalization for ocular disease.

i. Time to laboratory-confirmed COVID-19.

j. Patient adherence, defined as proportion correctly vaccinated for at least one dose or maintaining not vaccinated per protocol throughout the trial.

k. Proportion of vaccinated type and dose.

l. Proportion of uveitis remission throughout the trial.

Uveitis remission is defined as an inactive disease for 3 months after discontinuing all treatments for eye disease.

m. Vaccine inaccessibility, defined as proportion having gone to the vaccination site but refused due to the lack of vaccines, whereby this situation continues until the end of trial.

n. Reasons for not yet vaccinated until the end of trial among those who should have been vaccinated per protocol.

o. Change in anterior chamber cells from the Baseline to the end of three months.

p. Change in vitreous haze from the Baseline to the end of three months.

q. Change in best corrected visual acuity LogMAR score from the Baseline to the end of three months.

r. Incidence of any systemic adverse event (apart from uveitis related events).

4 Statistical Hypothesis

There is one formal comparison of interest on primary outcome:

Prompt vaccination strategy vs. Deferred vaccination strategy

Prompt vaccination strategy is hypothesized to result in an earlier occurrence of primary endpoint event as compared with deferred vaccination strategy.

The null hypothesis, that prompt vaccination strategy dose not result in an earlier occurrence of primary endpoint event, will be rejected if the estimated 95% confidence interval for the hazard ratio of prompt vaccination strategy lies wholly above 1.

5 Sample Size

A two-sided log-rank test with an overall sample size of 1314 subjects (657 in the deferred vaccination group and 657 in the prompt vaccination group) achieves 80.0% power at a 0.050 significance level to detect a hazard ratio of 1.5 when the proportion surviving (no occurrence of symptomatic uveitis worsening) in the deferred vaccination group is 0.9000. The study lasts for 6 time periods of which subject accrual (entry) occurs in the first 3 time periods. The accrual pattern across time periods is uniform (all periods equal). The proportion dropping out of both groups is 0.1500, respectively.

6 Randomization and Masking

Randomization will be designed to yield expected assignment ratio of 1:1 to the prompt vaccination strategy and the deferred vaccination strategy with the use of simple randomization. Randomization list will have been generated by a Statistician using a computer and will be properly kept by a Designated Study Coordinator. Both this Statistician and the Designated Study Coordinator will have no involvement in other parts of the trial. The Investigator who is designated as the Primary Treating Ophthalmologist and is responsible for the management of the subject in this trial will telephone the Designated Study Coordinator to obtain patient assignment information. Throughout the trial, the randomization assignment will not be masked to the Primary Treating Ophthalmologist, but the Primary Treating Ophthalmologist will not have direct access to the randomization list. The randomization assignment will be masked to an adjudication committee who will independently determine the study endpoint events.

7 Analysis Population

7.1 Intention-To-Treat Population

The Intent-To-Treat (ITT) Population will consist of all patients who have undergone randomization in the study regardless of their compliance with the study assignment. For all ITT analyses, participants will be analyzed as randomized.

7.2 Per Protocol Population

The Per Protocol Population will consist of eligible patients who have adhered to randomly assigned vaccination strategy and where study outcomes have been observed.

8 Missing Data

For baseline characteristics, missing values will be expected to be rare, and will not be imputed. In the primary analysis, the follow-up data will be censored at the follow-up encounter reporting the occurrence of primary endpoint event, withdrawal or loss to follow-up, or the final (month 3) visit (whichever is earlier). There is plan to impute missing data for those who withdraw or are lost to telephone follow up before month 3 with the use of multiple imputation in a sensitivity analysis. Analyses on secondary outcomes will be performed without data imputation.

9 Baseline Characteristic Analysis

A CONSORT flow diagram[1] will be used to summarize the randomization, treatment, adherence and follow-up of participants.

In general, baseline characteristics will be presented descriptively in the intention-to-treat population. According to the numerical characteristics of the variables, if the data follow a normal distribution, the mean with standard deviation will be used for statistical description; if the data are skewed, the median with interquartile range will be used for statistical description. Categorical variables will be summarized as numbers with percentages. Tests of statistical significance will not be taken for baseline characteristics.

Descriptive summaries of clinical variables will include age, gender, ethnic group, uveitis etiological and anatomical classification, severity of uveitis (duration, flares in the past year, and best corrected visual acuity of low vision or blindness) and comorbidities.

10 Primary Outcome Comparison

The primary outcome, time to uveitis symptomatic worsening over the course of 3 months of follow-up, will be compared with the Log-rank test. If a participant does not experience the primary endpoint event, they will have their data censored at the encounter where withdrawal or loss to follow-up occurs, or the final (month 3) follow-up encounter (whichever is earlier). For those with primary endpoint event, the censoring time point will be at the specific telephone call encounter (month 1, month 2, or month 3) where the occurrence of primary endpoint event is reported. Follow-up data after month 3 will no longer be included in the analysis, if any. The multiplicative limit method (Kaplan-Meier) will be used to show the cumulative incidence of primary endpoint event. A proportional hazard model (univariate Cox regression) is used to calculate the hazard ratio as well as 95% CI. The proportional-hazards assumption will be checked by means of inspection of a log-log survival plot.

11 Analysis of Secondary Outcomes

11.1 General Principles

The analysis of secondary outcomes will be performed by using an Intent-To-Treat framework within populations whose secondary outcome measures are available. No formal statistical comparisons for the secondary outcomes are planned, and no adjustment for multiple comparisons will be performed. No inferences can be drawn regarding the secondary outcomes, and these results will be descriptively presented as point estimates with unadjusted 95% confidence intervals only.

11.2 Analytical Methods

a. Time to clinically-confirmed uveitis worsening.

A proportional hazard model (univariate Cox regression) will be used to analyze the time to clinically-confirmed uveitis worsening.

b. Proportion of each reason for symptomatic uveitis worsening.

Descriptive analysis will be used to report the proportions between groups. Difference between proportions as well as its associated 95% CI will be estimated with the Wilson score method.[2]

c. Proportion of symptomatic uveitis worsening.

Descriptive analysis will be used to report the proportions between groups. Difference between proportions as well as its associated 95% CI will be estimated with the Wilson score method.

d. Proportion of each reason for clinically-confirmed uveitis worsening.

Descriptive analysis will be used to report the proportions between groups. Difference between proportions as well as its associated 95% CI will be estimated with the Wilson score method.

e. Time to initiation of step-up treatment.

A proportional hazard model (univariate Cox regression) will be used to analyze the time to initiation of step-up treatment.

f. Time to treatment interruptions.

A proportional hazard model (univariate Cox regression) will be used to analyze the time to treatment interruptions.

g. Time to hospitalization for any reasons.

A proportional hazard model (univariate Cox regression) will be used to analyze

the time to hospitalization for any reasons.

h. Time to hospitalization for ocular disease.

   A proportional hazard model (univariate Cox regression) will be used to analyze the time to hospitalization for ocular disease.

i. Time to laboratory-confirmed COVID-19.

   A proportional hazard model (univariate Cox regression) will be used to analyze the time to laboratory-confirmed COVID-19.

j. Patient adherence, defined as proportion correctly vaccinated for at least one dose or maintaining not vaccinated per protocol throughout the trial.

   Descriptive analysis will be used to report the patient adherence between groups. Difference between proportions as well as its associated 95% CI will be estimated with the Wilson score method.

k. Proportion of vaccinated type and dose.

   Descriptive analysis will be used to report the proportion of vaccinated type and dose administered between groups. Difference between proportions as well as its associated 95% CI will be estimated with the Wilson score method.

l. Proportion of uveitis remission throughout the trial.

   Descriptive analysis will be used to report the proportion of uveitis remission between groups. Difference between proportions as well as its associated 95% CI will be estimated with the Wilson score method.

m. Vaccine inaccessibility, defined as proportion having gone to the vaccination site but refused due to the lack of vaccines, whereby this situation continues until the end of trial.

   Descriptive analysis will be used to report the proportion of vaccine

inaccessibility between groups. Difference between proportions as well as its associated 95% CI will be estimated with the Wilson score method.

n. Reasons for not yet vaccinated until the end of trial among those who should have been vaccinated per protocol.

   Descriptive analysis will be used to report the each reason for not yet vaccinated between groups. Difference between proportions as well as its associated 95% CI will be estimated with the Wilson score method.

o. Change in anterior chamber cells from the Baseline to the end of three months.

   Descriptive analysis will be used to report the proportion of two-grade increase in anterior chamber cells (a clinically meaningful change as described by the SUN working group)[3] from the Baseline to the end of three months between groups. Difference between proportions as well as its associated 95% CI will be estimated with the Wilson score method.

p. Change in vitreous haze from the Baseline to the end of three months.

   Descriptive analysis will be used to report the proportion of two-grade increase in vitreous haze (a clinically meaningful change as described by the SUN working group)[3] from the Baseline to the end of three months between groups. Difference between proportions as well as its associated 95% CI will be estimated with the Wilson score method.

q. Change in best corrected visual acuity LogMAR score from the Baseline to the end of three months.

   A generalized estimating equation (GEE) will be used analyze the least-squares mean changes by treatment group with accounting for the correlation between eyes of the same patient and baseline LogMAR score per eye. For non-numerical visual acuities, the following denotations will be used as previously described:[4] finger count (FC) is assigned as 1.7 LogMAR, hand movement (HM) as 2.0

LogMAR, light perception (LP) as 2.3 LogMAR, and no light perception (NLP) as 3.0 LogMAR. Otherwise, numerical visual acuities will be transformed with the formula: logMAR = - $\log_{10}$(visual acuity fraction).[3]

r. Incidence of any systemic adverse event (apart from uveitis related events).

Descriptive analysis will be used to report the incidence of any systemic adverse event.

12 Subgroup Analysis

Subgroup analysis for the primary outcome comparison will be performed on the ITT population. A pre-specified subgroup analysis will be performed to determine differences between assignment groups across subgroup variable defined by uveitis anatomical subtypes, etiology diagnosis classification, history of uveitis flare, age and sex. The subgroup analysis will be conducted by additionally including the treatment-by-subgroup interaction term in the Cox regression model. We will test for heterogeneity of treatment effect across the subgroups and report the corresponding P value for interaction. A two-sided P value of less than 0.05 on the Wald χ^2 test will be considered to indicate statistical significance for the interaction term. If necessary, additional subgroup analyses will be performed as appropriate by testing other subgroup variables but will be considered exploratory.

13 Sensitivity Analysis Evaluating Non-Response

It is expected that there will be missing data on the primary outcome status for those who withdraw or are lost to follow-up before the final month 3 follow-up encounter. It is assumed that the missing data will be missing completely at random. The multiple imputation will be employed to predict the missing status (occurrence of the primary endpoint event or not) of those participants when outcome data are missing.[5] This analysis includes all ITT population. It is planned to predict the missing values in 20

imputation datasets based on all available baseline clinical characteristics, treatment assignment and completed follow-up encounters. The imputed outcome will be analyzed in each imputation dataset with the Cox regression model as it is performed in the primary analysis. With the use of the Rubin's rules, the results of the imputations will be combined to get a final answer that takes into consideration of the variability in the datasets.

14 Interim Analysis

This study does not plan to perform interim analysis.

15 Statistics Software

IBM SPSS Statistics, version 25.0 software will be primarily used for statistical analysis.

16 References

1. Moher D, Schulz KF, Altman DG. The CONSORT statement: revised recommendations for improving the quality of reports of parallel-group randomised trials. Lancet 2001;357:1191-4.

2. Newcombe RG. Two-sided confidence intervals for the single proportion: comparison of seven methods. Stat Med 1998;17:857-72.

3. Jabs D, Nussenblatt R, Rosenbaum J, Standardization of Uveitis Nomenclature (SUN) Working Group. Standardization of uveitis nomenclature for reporting clinical data. Results of the first international workshop. Am J Ophthalmol 2005;140:509-16.

4. Ch'ng SW, Patton N, Ahmed M, et al. The Manchester Large Macular Hole Study: Is it Time to Reclassify Large Macular Holes? Am J Ophthalmol 2018;195:36-42.

5. Harel O, Zhou XH. Multiple imputation: review of theory, implementation and software. Stat Med 2007;26:3057-77.
