# Supplementary material for: Early vs Deferred Non–Messenger RNA COVID-19 Vaccination Among Chinese Patients With a History of Inactive Uveitis: A Randomized Clinical Trial
Source: JAMA Netw Open. 2023 Feb 14;6(2):e2255804. doi: 10.1001/jamanetworkopen.2022.55804 (PMC9929699; doi:10.1001/jamanetworkopen.2022.55804)
Supplement: Supplement 2. — eMethods. Instrumental Variable Analyses eTable 1. Definition of Analysis Population eTable 2. Summary of Sensitivity Analyses eTable 3. As-Treated Analysis of Primary Outcome eTable 4. Reasons for Nonadherence to Vaccine Uptake as Recommended eTable 5. Subgroup Analysis of Primary Outcome eTable 6. Results of Sensitivity Analyses of Primary Outcome eTable 7. Instrumental Variable Analysis eTable 8. Comparative Analysis of Primary End Point Event Among Unvaccinated Participants eTable 9. Per Protocol Analysis of Ocular Condition at Month 3 in In-Person Evaluable Population eTable 10. Inverse Probability Weighting for Evaluating Ocular Condition at Month 3 to Account for Missing Data eTable 11. Definition of Systemic Adverse Events eTable 12. Summary of Systemic Adverse Events eFigure. Log-log Survival Plot for Primary Outcome eReferences [file jamanetwopen-e2255804-s002.pdf]

Supplementary Online Content

Zhong Z, Wu Q, Lai Y, et al. Early vs deferred non–messenger RNA COVID-19 vaccination among Chinese patients with a history of inactive uveitis: a randomized clinical trial. *JAMA Netw Open*. 2023;6(2):e2255804. doi:10.1001/jamanetworkopen.2022.55804

eMethods. Instrumental Variable Analyses
eTable 1. Definition of Analysis Population
eTable 2. Summary of Sensitivity Analyses
eTable 3. As-Treated Analysis of Primary Outcome
eTable 4. Reasons for Nonadherence to Vaccine Uptake as Recommended
eTable 5. Subgroup Analysis of Primary Outcome
eTable 6. Results of Sensitivity Analyses of Primary Outcome
eTable 7. Instrumental Variable Analysis
eTable 8. Comparative Analysis of Primary End Point Event Among Unvaccinated Participants
eTable 9. Per Protocol Analysis of Ocular Condition at Month 3 in In-Person Evaluable Population
eTable 10. Inverse Probability Weighting for Evaluating Ocular Condition at Month 3 to Account for Missing Data
eTable 11. Definition of Systemic Adverse Events
eTable 12. Summary of Systemic Adverse Events
eFigure. Log-log Survival Plot for Primary Outcome
eReferences

This supplementary material has been provided by the authors to give readers additional information about their work.

© 2023 Zhong Z et al. *JAMA Network Open*.

eMethods. Instrumental Variable Analyses

The instrumental variable analysis was used to assess the actual effect of COVID-19 vaccination on the primary outcome. The randomization assignment (early vs deferred COVID-19 vaccination) was considered a valid instrumental variable based on the assumption that it affected the outcome only by modifying a person's probability of receiving COVID-19 vaccines and was otherwise unrelated to measured or unmeasured confounders for the outcome. The instrumental variable analysis tested the actual effect by first generating via linear regression that early vaccination recommendation increased COVID-19 vaccine exposure (β1). The assessment of effect of early vaccination recommendation on the primary outcome was obtained from the primary analysis (β2). Under the assumption that the entire effect of early vaccination recommendation on the primary outcome (β2) was mediated by its effect on increasing COVID-19 vaccine exposure (β1), the assessment of causal effect on the primary outcome per increase in COVID-19 vaccine exposure was obtained by β2/β1. For the purposes of our study, we defined the exposure in two separate analyses. In analysis 1, the exposure was defined as receipt of COVID-19 vaccine during the study period. In analysis 2, the exposure was defined as having been vaccinated in line with the early vaccination recommendation during the study period. The difference was that two vaccinated individuals, who were assigned to receive the deferred vaccination recommendation and achieved complete uveitis remission to be vaccinated, were not categorized into those who have been vaccinated in line with the early vaccination recommendation. In both analysis 1 and analysis 2, we defined the outcome as the time to uveitis symptomatic worsening, which was the primary outcome of this study. To assess the causal effect on the primary outcome per increase in each exposure, we used the two-stage estimation approach for analyzing time-to-event data to estimate the hazard ratio as well as its 95% CI as previously described.[1,2]

© 2023 Zhong Z et al. *JAMA Network Open*.

eTable 1. Definition of Analysis Population

**Analysis Population**	**Sample Size**	**Definition**
Intention-to-treat	543	The population included all participants who had undergone randomization.
Modified Intention-to-treat	511	The population included all participants who met eligibility criteria and had undergone randomization. A total of 32 participants who had been vaccinated before randomization were excluded from the intention-to-treat population.
Per protocol	351	The population included participants who indeed followed randomly assigned vaccination recommendation (early or deferred) with follow-up data and had no vaccination before randomization. A total of 160 individuals who did not adhere to randomly assigned recommendation were excluded from the modified intention-to-treat population.
Tele-follow-up completed population	506	The population included participants who completed month 3 follow-up by telephone call. A total of 37 participants who withdrew or were lost to telephone call follow-up before month 3 were excluded from the intention-to-treat population.
Month-3 in-person evaluable population	249	The population included participants who had completed month 3 in-person follow-up encounter. A total of 294 participants who did not attend month 3 in-person follow-up visit were excluded from the intention-to-treat population.

© 2023 Zhong Z et al. *JAMA Network Open*.

eTable 2. Summary of Sensitivity Analyses

**Analysis**	**Summary**
Multiple imputation	To examine the potential impact of under-reporting of events due to withdrawal or loss to follow-up, multiple imputations were used to predict missing values in 20 imputed datasets for primary outcome of those who withdrew or were lost to tele-follow up before month 3 in the intention-to-treat population, based on the assumption that data were missing at random. Information about the recommendation allocation, completed follow-up encounters and all available values of baseline variables (age, sex, ethnic group, history of uveitis, etiology of uveitis, type of uveitis, best corrected visual acuity in the better seeing eye, number of flares in the past 12 months and medical history and comorbidities) was used for multiple imputations. The overall treatment effect hazard ratio was calculated by combining effects estimated from each imputed dataset in the Cox regression model with the Rubin's rule.
Multivariable Cox model*	Adjustment for known covariates may lead to increases in power.[3] Therefore, a Cox model as specified for the primary analysis in the intention-to-treat population was fit with further adjustment for all available baseline variables (age, sex, ethnic group, history of uveitis, etiology of uveitis, type of uveitis, best corrected visual acuity in the better seeing eye, number of flares in the past 12 months and medical history and comorbidities) in the sensitivity analysis.
Endpoint criteria modified*	In the sensitivity analysis, the endpoint criteria were modified and outcomes were re-adjudicated in the intention-to-treat population. Symptomatic uveitis worsening was re-defined if one of following newly onset symptoms occurred in at least one eye lasting for at least 2 days: eye redness, eye pain, decreased vision, or light sensitivity. Criterion of floaters was excluded to examine whether the results were sensitive to such a change in endpoint criteria.
Competing risks model*	Because a systemic event (eg. death or an adverse event of certain degree of severity) was likely to preclude the occurrence of primary endpoint event or greatly alter the chances to observe it, the competing-risks model (cumulative incidence function and Fine-Gray regression model) was used to account for the competing risk of systemic events in the intention-to-treat population.

* These analyses were performed post-hoc.

© 2023 Zhong Z et al. *JAMA Network Open*.

eTable 3. As-Treated Analysis of Primary Outcome

**Comparative Group**	**Total No.**	**No. Event**	**Cumulative incidence**	**Hazard Ratio (95% CI)**
**Primary analysis**				
Early vaccination recommendation	262	51	19.5%	1.68 (1.09-2.59)
Deferred vaccination recommendation	281	34	12.1%	1 [Reference]
**Analysis according to vaccination recommendation actually adhered to**				
Early vaccination	121*	29	24.0%	1.76 (1.13-2.76)
Deferred vaccination	422	56	13.3%	1 [Reference]
**Analysis according to vaccination status before the end of 3 months**				
Vaccinated with one dose or more	123*	29	23.6%	1.73 (1.10-2.70)
Not vaccinated with any dose	420	56	13.3%	1 [Reference]
**Analysis according to vaccine type administered during 3 month follow-up**				
Inactivated, CoronaVac (Sinovac)	68	16	23.5%	1.74 (1.00-3.03)
Inactivated, BBIBP-CorV (Sinopharm)	50	11	22.0%	1.59 (0.84-3.04)
Others†	5	2	40.0%	2.87 (0.70-11.76)
Not vaccinated	420	56	13.3%	1 [Reference]
**Analysis according to vaccine dose administered during 3 month follow-up**				
One dose	21	7	33.3%	2.56 (1.17-5.62)
Two doses	99	20	20.2%	1.46 (0.88-2.44)
Three doses	3	2	66.7%	4.96 (1.21-20.33)
Not vaccinated	420	56	13.3%	1 [Reference]

* Two vaccinated patients were not categorized into those who adhered to early vaccination recommendation because they were randomly assigned to the deferred vaccination

© 2023 Zhong Z et al. *JAMA Network Open*.

recommendation group and achieved complete uveitis remission to be vaccinated in line with the deferred vaccination recommendation.
† Two patients were vaccinated with an inactivated Vero-cell SARS-CoV-2 vaccine (Biokangtai, Shenzhen, China). Three patients were vaccinated with a protein subunit vaccine, ZF2001 (Zhifei, Chongqing China).

© 2023 Zhong Z et al. *JAMA Network Open*.

eTable 4. Reasons for Nonadherence to Vaccine Uptake as Recommended

**Variable**	**Early Vaccination Recommendation**	**Deferred Vaccination Recommendation**
No. of patients who should be vaccinated according to recommendation (advised to get vaccination during the trial period)	262	4
No. of patients who were not vaccinated as recommended (%)	153 (58.4)	2 (50.0)
Reasons for nonadherence, no./total no.(%)		
Not willing to get vaccinated (unspecified)	74/153 (48.4)	2/2 (100)
Not willing to get vaccinated (expression of willingness to get vaccinated after uveitis remission)	47/153 (30.7)	-
Willing to get vaccinated but having not yet gone to the vaccination site	2/153 (1.3)	-
Having gone to the vaccination site but refused due to contraindications judged by on-site physicians	9/153 (5.9)	-
Having gone to the vaccination site but refused due to the lack of vaccines (vaccine inaccessibility)	1/153 (0.7)	-
Following other clinicians' recommendation not to be vaccinated	2/153 (1.3)	-
Being in an acute phase of other diseases	1/153 (0.7)	-
Not willing to provide reasons or no reasons provided	17/153 (11.1)	-

© 2023 Zhong Z et al. *JAMA Network Open*.

eTable 5. Subgroup Analysis of Primary Outcome

Subgroup	Early Vaccination Recommendation	Deferred Vaccination Recommendation	Hazard Ratio* (95% CI)	P value for interaction
	no. with primary endpoint event/total no. (%)			
**Overall**	51/262 (19.5)	34/281 (12.1)	1.68 (1.09-2.59)	
**Age, year**				
≤16	2/16 (12.5)	2/30 (6.7)	2.19 (0.31-15.52)	0.779
>16	49/246 (19.9)	32/251 (12.7)	1.61 (1.03-2.52)	
**Sex**				
Female	26/149 (17.4)	20/155 (12.9)	1.44 (0.81-2.59)	0.455
Male	25/113 (22.1)	14/126 (11.1)	2.02 (1.05-3.89)	
**Uveitis anatomical classification**				
Anterior uveitis	16/74 (21.6)	13/71 (18.3)	1.25 (0.60-2.59)	0.345
Intermediate, posterior or panuveitis	35/188 (18.6)	21/210 (10.0)	1.93 (1.13-3.32)	
**Etiology of uveitis**				
Non-infectious	51/244 (20.9)	29/265 (10.9)	2.01 (1.28-3.17)	0.957
Infectious	0/18 (0.0)	5/16 (31.3)	Not estimated	
**No. of flares in the past 12 months†**				
0	30/194 (15.5)	22/205 (10.7)	1.52 (0.87-2.63)	0.551
≥1	21/68 (30.9)	12/76 (15.8)	2.02 (1.00-4.11)	

* The hazard ratio is shown for the primary endpoint event, symptomatic uveitis worsening.

† Missing data of the variable were imputed as zero for four patients.

© 2023 Zhong Z et al. *JAMA Network Open*.

eTable 6. Results of Sensitivity Analyses of Primary Outcome

**Analysis**	**Early Vaccination Recommendation**	**Deferred Vaccination Recommendation**	**Hazard Ratio*** **(95% CI)**
	no. with primary endpoint event/total no.(%)		
Primary analysis	51/262 (19.5)	34/281 (12.1)	1.68 (1.09-2.59)
Multiple imputation	NA	NA	1.63 (1.07-2.47) †
Multivariable Cox model	51/262 (19.5)	34/281 (12.1)	1.60 (1.02-2.51)
Endpoint criteria modified	47/262 (17.9)	34/281 (12.1)	1.55 (0.99-2.40)
Competing risks model	51/262 (19.5)	34/281 (12.1)	1.68 (1.10-2.55)

Abbreviations: NA, not applicable

* The hazard ratio is shown for the primary endpoint event, symptomatic uveitis worsening.

† The hazard ratio and the 95% CI were combined with the Rubin's rule from 20 imputation datasets.

© 2023 Zhong Z et al. *JAMA Network Open*.

eTable 7. Instrumental Variable Analysis

**Effect**	**Reference**	**Outcome**	**Effect Estimate (Coefficient± Standard Error)**	**Hazard Ratio (95% CI)**
**Instrumental variable analysis 1***				
Early vaccination recommendation	Deferred vaccination recommendation	Time to uveitis symptomatic worsening	0.518±0.221	1.68 (1.09-2.59)
Early vaccination recommendation	Deferred vaccination recommendation	Proportion of vaccination	0.366±0.032	Not applicable
**Vaccination**	**No vaccination**	**Time to uveitis symptomatic worsening**	**1.415±0.605**	**4.12 (1.26-13.46)**
**Instrumental variable analysis 2***				
Early vaccination recommendation	Deferred vaccination recommendation	Time to uveitis symptomatic worsening	0.518±0.221	1.68 (1.09-2.59)
Early vaccination recommendation	Deferred vaccination recommendation	Proportion of early vaccination	0.373±0.032	Not applicable
**Early vaccination**	**Deferred vaccination**	**Time to uveitis symptomatic worsening**	**1.381±0.594**	**3.98 (1.24-12.75)**

* These analyses aimed to assess the effect of vaccination (analysis 1) and the per protocol effect (analysis 2) on the primary outcome. In analysis 1, the exposure was defined as receipt of COVID-19 vaccine during the study period. In analysis 2, the exposure was defined as having been vaccinated in line with the early vaccination recommendation. The difference was that two vaccinated individuals, who were assigned to the deferred vaccination recommendation and achieved complete uveitis remission to be vaccinated, were not categorized into those who have been vaccinated in line with the early vaccination recommendation. Both outcomes were the time to uveitis symptomatic worsening. The randomization assignment (early versus deferred vaccination recommendation) was considered a valid instrumental variable based on the assumption that it affected the outcome only by influencing a person's probability of receiving COVID-19 vaccine and was otherwise unrelated to measured and unmeasured confounders for the outcome. The estimate of hazard ratio as well as its 95% CI was obtained with the use of the two-stage estimation approach for analyzing time-to-event data as previously described.[1,2]

© 2023 Zhong Z et al. *JAMA Network Open*.

eTable 8. Comparative Analysis of Primary End Point Event Among Unvaccinated Participants

Group*	Total No.	No. Event	Cumulative incidence	Hazard Ratio (95% CI)
Early vaccination recommendation	153	23	15.0%	1.30 (0.77-2.22)
Deferred vaccination recommendation	267	33	12.4%	1 [Reference]

* This analysis compared the time to primary endpoint event between randomly assigned groups in participants who had not been vaccinated before the end of trial.

© 2023 Zhong Z et al. *JAMA Network Open*.

eTable 9. Per Protocol Analysis of Ocular Condition at Month 3 in In-Person Evaluable Population

**Outcome**	**Early Vaccination Recommendation**	**Deferred Vaccination Recommendation**	**Difference (95% CI)**
Per protocol & in-person evaluable population*	39	126	-
Two-grade increase in anterior chamber cells, no./total no. (%)†	5/39 (12.8)	12/126 (9.5)	3.3% (-6.3 to 17.7)
Two-grade increase in vitreous haze, no./total no. (%)†	1/39 (2.6)	2/126 (1.6)	1.0% (-3.6 to 11.7)
Change in best corrected visual acuity, LogMAR‡	0.012±0.017	0.004±0.017	0.008 (-0.039 to 0.557)

* A total of 165 participants overlapped in both per protocol and in-person evaluable population.
† Increases were relative to baseline condition.
‡ Changes in best corrected visual acuity were analyzed by eye with the generalized estimating equation to account for baseline values and the correlation between eyes of the same patient. Data are shown as least-squares means ± standard errors. Visual acuity data are expressed as scores for the log of the minimum angle of resolution (logMAR), with higher values indicating poorer vision.

© 2023 Zhong Z et al. *JAMA Network Open*.

eTable 10. Inverse Probability Weighting for Evaluating Ocular Condition at Month 3 to Account for Missing Data

**Outcome**	**Early Vaccination Recommendation**	**Deferred Vaccination Recommendation**	**Difference (95% CI)**
Inverse probability weighted pseudopopulation*	247	290	-
Two-grade increase in anterior chamber cells, no./total no. (%)†	18/247 (7.3)	26/290 (9.0)	-1.7% (-6.3 to 3.1)
Two-grade increase in vitreous haze, no./total no. (%)†	2/247 (0.8)	5/290 (1.7)	-0.9% (-3.2 to 1.4)
Change in best corrected visual acuity, LogMAR‡	-0.006±0.007	-0.001±0.011	-0.005 (-0.029 to 0.020)

* The pseudopopulation was generated with the use of inverse probability weighting to account for unobserved data of participants who were not included in the month-3 in-person evaluable population. The probability of non-missing information at month 3 was predicted with the logistic regression model, where the response was the nonmissingness and the covariates included the occurrence of primary endpoint event during study period and all available values of baseline variables (age, sex, ethnic group, history of uveitis, etiology of uveitis, type of uveitis, best corrected visual acuity in the better seeing eye, number of flares in the past 12 months and medical history and comorbidities). The weight of each participant was given by the inverse of the predicted probability. Then, the analysis was performed only on the non-missing observations of the month-3 in-person evaluable population with a weighted model. Numbers of valid cases have been rounded.

† Increases were relative to baseline condition.

‡ Changes in best corrected visual acuity were analyzed by eye with the generalized estimating equation to account for baseline values and the correlation between eyes of the same patient. Data are shown as least-squares means ± standard errors. Visual acuity data are expressed as scores for the log of the minimum angle of resolution (logMAR), with higher values indicating poorer vision.

© 2023 Zhong Z et al. *JAMA Network Open*.

eTable 11. Definition of Systemic Adverse Events

**Event**	**Definition**
Death	Death
Pneumonia	A disorder characterized by inflammation focally or diffusely affecting the lung parenchyma
Upper respiratory infection	A disorder characterized by an infectious process involving the upper respiratory tract (nose, paranasal sinuses, pharynx, larynx, or trachea
Tachycardia	A heart rhythm disorder characterized by heartbeats faster than usual, greater than 100 beats per minute
Hypertension	A disorder characterized by a pathological increase in blood pressure
Stomach pain	A disorder characterized by a sensation of marked discomfort in the stomach
Irregular menstruation	A disorder characterized by a change in cycle or duration of menses from baseline
Vaginal infection	A disorder characterized by an infectious process involving the vagina
Arthralgia	A disorder characterized by a sensation of marked discomfort in a joint
Fatigue	A disorder characterized by a state of generalized weakness with a pronounced inability to summon sufficient energy to accomplish daily activities
Dizziness	A disorder characterized by a disturbing sensation of lightheadedness, unsteadiness, giddiness, spinning or rocking

© 2023 Zhong Z et al. *JAMA Network Open*.

eTable 12. Summary of Systemic Adverse Events

**Event**	**Early Vaccination Recommendation (N = 262) ***	**Deferred Vaccination Recommendation (N = 281) ***	**Severity**	**Temporal relation**
Death	0	1 (0.4)	Severe -life threatening	Event before vaccination
Pneumonia	0	2 (0.7)	Severe - hospitalized	Both events before vaccination
Upper respiratory infection	2 (0.8)	2 (0.7)	Moderate	All events before vaccination
Tachycardia	0	1 (0.4)	Mild	Event before vaccination
Hypertension	0	1 (0.4)	Moderate	Event before vaccination
Stomach pain	1 (0.4)	0	Moderate	Event before vaccination
Irregular menstruation	0	1 (0.4)	Mild	Event before vaccination
Vaginal infection	0	1 (0.4)	Mild	Event before vaccination
Arthralgia	0	1 (0.4)	Mild	Event before vaccination
Fatigue	1 (0.4)	0	Mild	Event before vaccination
Dizziness	0	1 (0.4)	Mild	Event before vaccination

* Data are shown as no. (%).

© 2023 Zhong Z et al. *JAMA Network Open*.

eFigure. Log-log Survival Plot for Primary Outcome

Visual inspection of the log-log survival plot suggested that the proportional-hazards assumption was met.

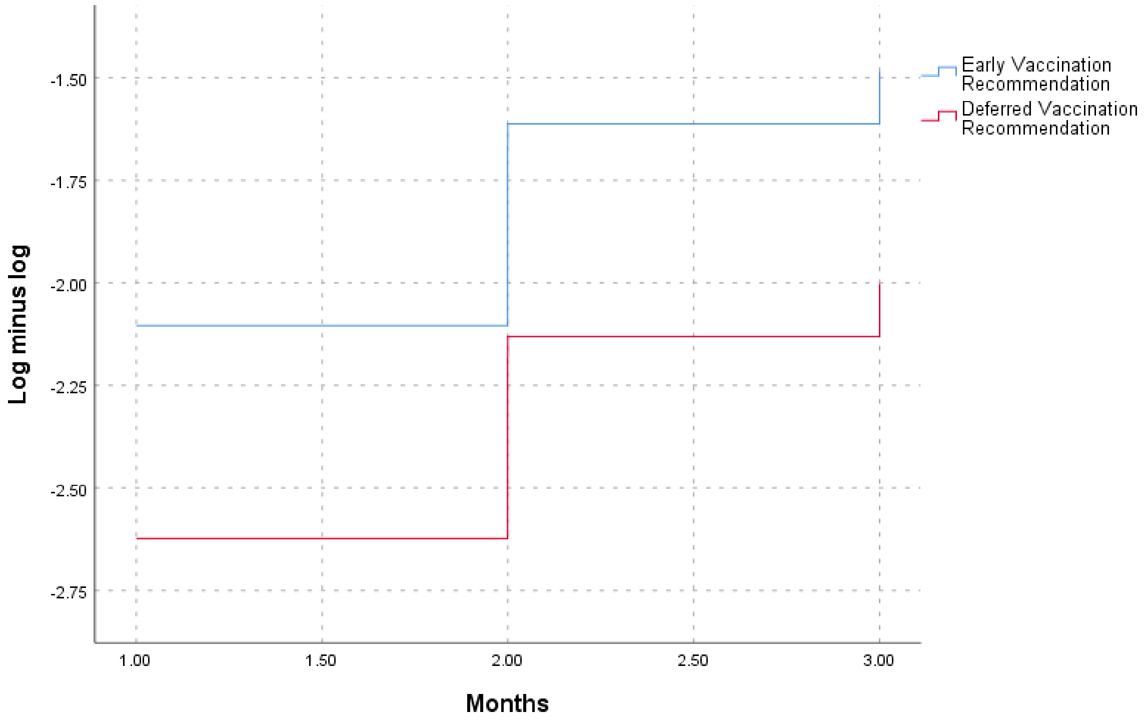

© 2023 Zhong Z et al. *JAMA Network Open*.

eReferences

1. Tchetgen Tchetgen EJ, Walter S, Vansteelandt S, Martinussen T, Glymour M. Instrumental variable estimation in a survival context. *Epidemiology.* 2015;26(3):402-410.
2. Sjolander A, Martinussen T. Instrumental Variable Estimation with the R Package ivtools. *Epidemiologic Methods.* 2019;8(1):1-20.
3. Kahan BC, Jairath V, Doré CJ, Morris TP. The risks and rewards of covariate adjustment in randomized trials: an assessment of 12 outcomes from 8 studies. *Trials.* 2014;15(139):1745-6215.

© 2023 Zhong Z et al. *JAMA Network Open*.
