# Supplementary material for: Early vs Deferred Non–Messenger RNA COVID-19 Vaccination Among Chinese Patients With a History of Inactive Uveitis: A Randomized Clinical Trial
Source: JAMA Netw Open. 2023 Feb 14;6(2):e2255804. doi: 10.1001/jamanetworkopen.2022.55804 (PMC9929699; doi:10.1001/jamanetworkopen.2022.55804)
Supplement: Supplement 3. — Data Sharing Statement [file jamanetwopen-e2255804-s003.pdf]

Data Sharing Statement

Zhong. Early vs Deferred Non-Messenger RNA COVID-19 Vaccination Among Chinese Patients With a History of Inactive Uveitis. *JAMA Netw Open*. Published February 14, 2023. doi:10.1001/jamanetworkopen.2022.55804

Data
Data available: Yes
Data types: Deidentified participant data
How to access data: Data will be accessed from the corresponding author (peizengycmu@126.com).
When available: With publication

Supporting Documents
Document types: None

Additional Information
Who can access the data: Researchers whose proposed use of the data has been approved.
Types of analyses: For a specified purpose.
Mechanisms of data availability: With investigator support, after approval of a proposal, and with a signed data access agreement.
Any additional restrictions: Data should not be used for commercial purpose.
